# Electroacupuncture alleviates Parkinson’s disease by targeting HDAC/SIRT-mediated deacetylation of 14-3-3

**DOI:** 10.3389/fnagi.2025.1719326

**Published:** 2026-01-14

**Authors:** Zhao-Qin Wang, Han-Dan Zheng, Ling-Jie Li, Lu-Lu Cao, Lin Shen, Yu Qiao, Yi-Yi Chen, Lu-Yi Wu, Guo-Na Li, Huan-Gan Wu

**Affiliations:** 1 Yueyang Hospital of Integrated Traditional Chinese and Western Medicine, Shanghai University of Traditional Chinese Medicine, Shanghai, China; 2 Shanghai Research Institute of Acupuncture and Meridian, Shanghai, China; 3 Shanghai Municipal Hospital of Traditional Chinese Medicine, Shanghai University of Traditional Chinese Medicine, Shanghai, China

**Keywords:** 14-3-3 protein, acetylation, acupuncture, fecal microbiota transplantation, MPTP, Parkinson’s disease, Ywhaq

## Abstract

**Introduction:**

Parkinson’s disease (PD) is a neurodegenerative disorder characterized by the loss of dopaminergic neurons and the accumulation of pathological *α*-synuclein. Although current treatments can alleviate symptoms, they do not modify disease progression. Growing evidence implicates gut microbiota dysbiosis and aberrant protein acetylation in PD pathogenesis. Electroacupuncture (EA) has shown therapeutic potential in PD; however, its effects on protein acetylation remain unclear.

**Methods:**

A PD mouse model was established through MPTP induction and fecal microbiota transplantation (FMT) from PD patients. Mice received EA stimulation at *Baihui* (GV20) and *Yanglingquan* (GB34) acupoints for 14 days. Behavioral tests, immunohistochemistry, Western blot, qPCR, and 4D label-free acetyl proteomics were employed to assess motor function, neuronal integrity, protein expression, and acetylation profiles.

**Results:**

EA significantly improved motor coordination, enhanced sensorimotor function in the adhesive removal test, and increased open-field activity in PD mice. It attenuated the loss of tyrosine hydroxylase–positive neurons and decreased *α*-synuclein accumulation in the substantia nigra. Proteomic analysis revealed hyperacetylation of Ywhaq (14-3-3) in PD mice, which was reversed by EA. Mechanistically, EA upregulated the expression of deacetylases HDAC1/2/3 and SIRT1/2 at both protein and mRNA levels, restoring acetylation homeostasis.

**Conclusion:**

Electroacupuncture ameliorates behavioral and neuropathological phenotypes in a PD mouse model by restoring deacetylase expression and normalizing protein acetylation, particularly of 14-3-3. Our results underscore the therapeutic potential of EA and highlight acetylation modulation as a promising strategy for PD treatment.

## 1 Introduction

Parkinson’s disease is the second most common neurodegenerative disorder, characterized by the progressive loss of dopaminergic neurons in the substantia nigra pars compacta and the presence of Lewy bodies containing aggregated *α*-synuclein (Wakabayashi et al., 2013; Surguchov and Surguchev, 2022). While motor symptoms such as bradykinesia, tremor, and rigidity are clinical hallmarks, non-motor manifestations including gastrointestinal dysfunction often precede motor decline by years, highlighting the involvement of the gut-brain axis in PD pathogenesis (Carabotti et al., 2015; Feng et al., 2024; Grotewold and Albin, 2024).

Emerging evidence indicates that gut microbiota dysbiosis contributes to PD development through multiple mechanisms, including immune activation, inflammation, and impaired barrier function (Feng et al., 2024; Sampson et al., 2016). Transplantation of fecal microbiota from PD patients into mice can induce motor deficits and neuroinflammation, supporting a causal role of gut microbes in PD (Sun et al., 2018). Additionally, recent studies have revealed that dysregulation of protein acetylation—a key post-translational modification—is implicated in neurodegenerative processes (Wang R. et al., 2020). Reduced activity of deacetylases such as HDACs and SIRTs leads to hyperacetylation of various proteins, disrupting cellular homeostasis and promoting neuronal death (Yakhine-Diop et al., 2019). Notably, short-chain fatty acids (SCFAs), gut microbiota-derived metabolites, can inhibit HDAC activity in multiple tissues including the brain (Koh et al., 2016). In PD patients, symptom severity is positively correlated with plasma SCFA levels (Chen et al., 2022). Thus, SCFAs may act as a critical link between gut dysbiosis and PD pathogenesis, possibly by modulating HDAC activity and contributing to protein acetylation dysregulation.

Electroacupuncture has been widely used in China and increasingly recognized globally as a complementary therapy for PD (Li et al., 2023). Clinical and experimental studies have demonstrated that EA can improve motor function, reduce *α*-synuclein aggregation, and modulate neuroinflammation (Li et al., 2025; Zeng and Zhao, 2016; Zhao et al., 2021; Yu et al., 2025). Our previous work showed that EA enhances gut microbial diversity and ameliorates PD pathology in mouse models (Wang Z. Q. et al., 2020), yet whether it influences protein acetylation remains unknown. In this study, we integrated MPTP-induced neurotoxicity with fecal microbiota transplantation from PD patients to establish a comprehensive PD mouse model that recapitulates both central and peripheral features of the disease. We aimed to determine whether EA attenuates motor deficits in an animal model of PD by modulating protein acetylation and to investigate the role of HDACs and SIRTs in this process. Our findings provide new insights into the mechanisms of EA and support its potential as a disease-modifying therapy for PD.

## 2 Materials and methods

### 2.1 Animals

A total of eighty-two specific pathogen-free (SPF) male C57BL/6 mice (8 weeks old, weighing 25–30 g) were supplied by Shanghai Lingchang Biotechnology. The animals were housed in the SPF facility of the Laboratory Animal Center at Shanghai University of Traditional Chinese Medicine under controlled conditions: temperature of 22–24 °C, humidity of 50–65%, and a 12-h light/dark cycle. All mice underwent 1 week of acclimatization before experiments. The experimental procedures were approved by the Animal Ethics Committee of Shanghai University of Traditional Chinese Medicine (approval No. PZSHUTCM220725004) July 25, 2022, and conducted in accordance with the National Institutes of Health Guide for the Care and Use of Laboratory Animals.

### 2.2 Donor screening and fecal microbiota transplantation (FMT) protocol

Fecal donors were recruited from outpatients at the Acupuncture Department of Yueyang Hospital of Integrated Traditional Chinese and Western Medicine, Shanghai University of Traditional Chinese Medicine, as well as through public advertisements. Based on their clinical status, donors were classified into two groups: the Parkinson’s disease fecal microbiota transplantation (PD-FMT) group and the healthy control fecal microbiota transplantation (HC-FMT) group. All procedures were approved by the Institutional Ethics Committee (Approval No. 2022-094).

#### 2.2.1 PD patient FMT donor selection criteria

PD patient donors of FMT were selected based on the following criteria: inclusion required a diagnosis of Parkinson’s disease consistent with the UK Parkinson’s Disease Society Brain Bank Diagnostic Criteria (1992), age between 40 and 80 years, and provision of written informed consent specifically for stool donation. Key exclusion criteria comprised atypical or secondary parkinsonian syndromes, history of malignancy or chronic gastrointestinal diseases such as inflammatory bowel disease or irritable bowel syndrome, significant comorbidities in major organ systems, presence of psychiatric disorders, recent use of probiotics, antibiotics, or NSAIDs within 3 months prior to donation, undergoing any invasive gastrointestinal procedure within the same period, and adherence to an exclusively vegetarian diet during the 3 months before enrollment.

#### 2.2.2 Healthy donor FMT screening criteria

Healthy FMT donors were matched to PD patients by age, sex, and education level whenever possible. Eligible donors were required to be between 40 and 80 years old, exhibit no clinically significant abnormalities upon physical examination and routine blood tests, and provide written informed consent specifically for stool donation. Furthermore, donors were rigorously screened to exclude individuals with any history or symptoms of neurological or digestive diseases, a family history of heritable or organic disorders, conditions known to alter gut microbiota composition—such as obesity (BMI ≥ 28 kg/m^2^)—as well as those who had used probiotics, antibiotics, NSAIDs, or any prescription medications within the 3 months preceding donation. Additional exclusions comprised having undergone invasive procedures or examinations during the same period, adherence to an exclusively vegetarian diet, and current pregnancy or lactation.

#### 2.2.3 Fecal sample collection

Fecal samples were collected from eligible PD patients and healthy control subjects. Prior to collection, clean ice packs were frozen overnight. During the sampling process, participants defecated into disposable sterile bedpans placed on toilet seats to avoid contamination with urine or other bodily fluids. Approximately 20 mL of mid-stream fecal material was collected using sterile disposable containers. Immediately after collection, samples were placed in pre-cooled insulated boxes containing ice packs and transported to the laboratory within 2 h. All samples were subsequently stored at −80 °C until further processing.

#### 2.2.4 Fecal suspension preparation

Fecal microbiota suspensions for FMT were prepared as described previously by Hou et al. (2021), with the specific protocol as follows: donor feces (mixed in equal weights) were diluted in sterile PBS to a concentration of 20 mg/mL. The fecal mixture was soaked in sterile PBS for approximately 15 min, vortexed thoroughly, and centrifuged at 1000 rpm at 4 °C for 5 min; the supernatant was then collected. Subsequent centrifugation was performed at 8000 rpm at 4 °C for 5 min to pellet the fecal microbiota, which was then filtered twice with sterile PBS. The resulting fecal microbiota suspension was mixed with an equal volume of 40% sterile glycerol and stored at −80 °C until use.

#### 2.2.5 Fecal microbiota transplantation protocol

Prior to FMT, mice were administered broad-spectrum antibiotics via drinking water for 4 weeks to deplete gut microbiota. The antibiotic regimen included ampicillin (1 g/L, A610028, Sangon Biotech), neomycin (1 g/L, A610366, Sangon Biotech), metronidazole (1 g/L, A600633, Sangon Biotech), and vancomycin hydrochloride (0.5 g/L, A100990, Sangon Biotech). Following antibiotic treatment, starting the next day, mice received intragastric administration of fecal microbiota suspension at a volume of 200 μL per mouse once daily for 4 consecutive weeks to establish the model. Mouse body weights were measured every other day, and general conditions were observed and recorded.

### 2.3 PD model establishment

Mice were randomly divided into a normal control group and a modeling group, comprising Healthy control fecal microbiota transplantation group and PD patient fecal microbiota transplantation group. Following the classical MPTP-induced PD model protocol by Jackson-Lewis and Przedborski (2007), modeling group mice received daily intraperitoneal injections of MPTP (30 mg/kg, M0896, Sigma) for 5 consecutive days (during the final 5 days of FMT). Body weight and general conditions were recorded daily. Four mice died during the modeling process. Post-modeling, two randomly selected modeling group mice and two normal control group mice underwent model validation via tyrosine hydroxylase (TH) immunohistochemical staining of the substantia nigra to confirm successful model establishment.

### 2.4 Animal grouping and intervention methods

After successful model establishment, the animals were divided into the following groups: the normal control (NC) group, which received handling and restraint identical to the treatment groups without therapeutic intervention; the healthy control fecal microbiota transplantation (HC-FMT) group, which also received handling and restraint without additional treatment; the PD patient fecal microbiota transplantation (PD-FMT) group, subjected to the same handling and restraint procedures; the electroacupuncture (EA) group, which received daily intervention starting 24 h post-modeling, involving gentle restraint in custom holders and needle insertion at *Baihui* (midline parietal bone) and *Yanglingquan* (anterior and inferior to the fibular head) using sterile disposable acupuncture needles (0.16 × 7 mm; Suzhou Huatuo)—with insertion depth of 4 mm, initial twirling for 1 min followed by repeated twirling every 5 min, and a total needle retention of 15 min per session, administered once daily for 14 consecutive days according to established protocols from *Experimental Acupuncture Science*; and the rasagiline (RAS) group, which received daily oral administration of rasagiline mesylate (0.2 mg/kg) via gavage for 14 days starting 24 h after modeling.

### 2.5 Rotarod test

The rotarod test was conducted using a commercially available apparatus (manufactured by TME, Chengdu, China) with a rod diameter of 2 cm. Mice were positioned on the rod with their heads facing perpendicular to its long axis. The rotational speed was gradually accelerated from 10 rpm to 30 rpm over a 5-min period. Before MPTP administration, all animals underwent a 3-day training session to acquire the ability to remain on the rod rotating at 20 rpm for at least 120 s. On the day after the final intervention, the latency to fall was recorded at five incremental speeds: 10, 15, 20, 25, and 30 rpm. An Overall Rotarod Performance (ORP) score for each group was derived using the trapezoidal method to integrate endurance across all speed levels.

### 2.6 The adhesive removal test

The adhesive removal test was used to assess the fine motor function (Bouet et al., 2009). A small adhesive tape patch was firmly attached to the glabrous skin of the mouse’s forepaw, covering the palmar surface including the paw pad, thenar, and hypothenar regions. Each mouse was then placed individually into its home cage, and the time required to first sense (initial contact) and fully remove the adhesive tape was recorded. Each animal underwent three trials, and the average time of successful removal was used for statistical analysis.

### 2.7 Open field test

The open field test was conducted in a rectangular arena measuring 27 × 27 × 30 cm (Deacon, 2006). Each mouse was placed individually in the center of the arena and allowed to explore freely for 5 min while its movement was recorded and tracked using a video tracking system. The arena was thoroughly cleaned with 75% ethanol and dried completely between trials to eliminate olfactory cues. Testing was performed in a quiet environment to minimize external disturbances.

### 2.8 Immunofluorescence staining

Immunofluorescence staining was performed to detect TH-positive neurons in the substantia nigra. Midbrain tissues from mice were fixed in 4% paraformaldehyde, embedded in paraffin, and coronally sectioned at a thickness of 4 μm. Sections were heated at 62 °C, deparaffinized with xylene, and rehydrated through a graded ethanol series. After antigen retrieval and blocking of endogenous peroxidase activity, the sections were incubated with 5% bovine serum albumin for 20 min at room temperature to prevent nonspecific binding. Subsequently, sections were incubated overnight at 4 °C with a primary antibody against TH (sc-25269, Santa Cruz Biotechnology; dilution 1:100). Following PBS washes, the sections were incubated with a fluorophore-conjugated secondary antibody for 30 min at 37 °C in the dark. Nuclei were counterstained with DAPI. After final washes, the sections were mounted with antifading mounting medium. Images were acquired using a fluorescence microscope, and the fluorescence intensity of TH-positive signals in the substantia nigra was quantitatively analyzed using ImageJ software.

### 2.9 Western blot analysis

The expression of *α*-synuclein, 14–3-3, HDAC1, HDAC2, HDAC3, SIRT1, and SIRT2 in the mouse substantia nigra were assessed by Western blot. For each group, 20 mg of midbrain tissue was homogenized, lysed, and centrifuged to collect the supernatant containing total protein. Protein concentration was determined using the BCA assay. Proteins were denatured by heating in a metal bath, and 20 μg of total protein per sample was separated by SDS-PAGE and transferred onto a PVDF membrane. The membrane was blocked with 3% non-fat milk for 1 h at room temperature, then incubated with primary antibodies against *α*-synuclein (ab212184, Abcam, 1:2000), 14–3-3 (SYZIA-R1, Sino Biological, 1:500), HDAC1 (ab109411, Abcam, 1:5000), HDAC2 (ab32117, Abcam, 1:2000), HDAC3 (ab32369, Abcam, 1:8000), SIRT1 (ab110304, Abcam, 1:4000), and SIRT2 (ab211033, Abcam, 1:2000) and *β*-Actin (1:80000) overnight at 4 °C. After washing, membranes were incubated with horseradish peroxidase (HRP)-conjugated secondary antibody (1:20000) for 1 h at room temperature. Following additional washes, protein bands were visualized using enhanced chemiluminescence and imaged with a gel imaging system. Band intensities were quantified using ImageJ software. The ratio of *α*-synuclein to β-Actin was calculated for each sample to normalize protein expression levels.

### 2.10 Quantitative real-time PCR (qPCR) analysis

Total RNA was extracted from substantia nigra tissues using TRIzol reagent (15,596,018, Invitrogen) with chloroform (BCP) extraction and isopropanol precipitation, followed by 75% ethanol washing and air-drying. RNA was quantified using spectrophotometer after 5-fold dilution. Only samples with ratios between 1.8 and 2.0 and greater than 2.0 respectively, were used for downstream analysis. To confirm the absence of genomic DNA contamination, we performed agarose gel electrophoresis on representative RNA samples prior to reverse transcription. The presence of clear, sharp 28S and 18S ribosomal RNA bands without visible genomic DNA smearing confirmed high RNA integrity and the absence of significant DNA contamination. cDNA synthesis was performed from 2 μg total RNA using random primers and RevertAid reverse transcriptase (25 °C for 5 min, 42 °C for 60 min). qPCR was conducted using SYBR Green Master Mix (208,054, QIAGEN) with gene-specific primers (Table 1) on Real-Time PCR System, with 10 μL reaction volume (1.5 μL H_2_O, 5 μL 2 × SYBR Green PCR Master Mix, 1 μL 10 μM primers, 2.5 μL cDNA template) and thermal cycling conditions of 95 °C for 2 min, followed by 45 cycles of 95 °C for 5 s and 60 °C for 10 s. Relative gene expression was normalized to GAPDH using 2^−ΔΔCt^ methods.

**Table 1:** The primer sequences of HDAC1, HDAC2, HDAC3, SIRT1, SIRT2, and GAPDH.

Gene	Primer sequence (5′-3′)
mouse GAPDH Forward	aggtcggtgtgaacggatttg
mouse GAPDH Reverse	tgtagaccatgtagttgaggtca
mouse hdac1 F-primer	gaccctgacaaacgcatctc
mouse hdac1 R-primer	ccaccttctccctcctcatc
mouse hdac2 F-primer	gttttgtcagctctccacgg
mouse hdac2 R-primer	aattcgaggatggcaagcac
mouse hdac3 F-primer	acactgtccgaaatgttgcc
mouse hdac3 R-primer	tggggcaaagtactcgaagt
mouse sirt1 F-primer	ctggggtttctgtctcctgt
mouse sirt1 R-primer	aggcgagcatagataccgtc
mouse sirt2 F-primer	gcagaacatagacacgctgg
mouse sirt2 R-primer	ctttcatccagcccatcgtg

### 2.11 4D label-free acetyl proteomic analysis

Comprehensive 4D label-free acetyl proteomic analysis was performed to profile global protein acetylation. Detailed experimental procedures—including sample preparation, LC–MS/MS instrumentation parameters, data acquisition, and bioinformatic processing—are provided in the Additional File 1. Bioinformatics analyses included Gene Ontology (GO) enrichment, KEGG pathway annotation, subcellular localization prediction, and COG/KOG functional classification.

#### 2.11.1 Protein extraction

Frozen midbrain tissues were pulverized in liquid nitrogen. The resulting powder was lysed in four volumes of buffer (8 M urea, 1% protease inhibitor cocktail, 3 μM TSA, 50 mM nicotinamide) followed by sonication. Cellular debris was removed by centrifugation at 12,000 × g for 10 min at 4 °C. The supernatant was collected, and protein concentration was determined using a BCA assay.

#### 2.11.2 Trypsin digestion

Proteins were precipitated with 20% trichloroacetic acid for 2 h at 4 °C, washed with ice-cold acetone, and air-dried. The pellet was resuspended in 200 mM TEAB. Proteins were reduced with 5 mM dithiothreitol at 56 °C for 30 min, alkylated with 11 mM iodoacetamide at room temperature for 15 min in the dark, and digested overnight with trypsin at a 1:50 (w/w) enzyme-to-protein ratio.

#### 2.11.3 Acetyllysine peptide enrichment

Tryptic peptides were incubated overnight at 4 °C with gentle shaking using anti-acetyllysine antibody-conjugated beads (PTM-104, PTM Bio) in immunoprecipitation buffer (100 mM NaCl, 1 mM EDTA, 50 mM Tris–HCl, 0.5% NP-40, pH 8.0). Beads were washed with IP buffer and deionized water, and bound peptides were eluted with 0.1% trifluoroacetic acid. Eluates were desalted using C18 ZipTips and lyophilized.

#### 2.11.4 LC-MS/MS analysis

Peptides were separated on a nanoElute UHPLC system with a 60-min gradient from 6 to 80% mobile phase B (A: 0.1% formic acid in 2% acetonitrile; B: 0.1% formic acid in 100% acetonitrile) at a flow rate of 450 nL/min. Eluted peptides were ionized at 1.7 kV and analyzed using a timsTOF Pro mass spectrometer operated in PASEF mode. Full MS scans (m/z 100–1700) were acquired, followed by up to 10 MS/MS scans per cycle with dynamic exclusion set to 30 s.

#### 2.11.5 Data processing

Raw data were processed using MaxQuant (v1.6.15.0) against the UniProt *Mus musculus* database supplemented with decoy and contaminant entries. Search parameters included: trypsin/P digestion with up to 4 missed cleavages allowed; minimum peptide length of 7 amino acids; maximum of 5 modifications per peptide; mass tolerances of 20 ppm for both precursor and fragment ions. Fixed modification was carbamidomethylation (C), and variable modifications included oxidation (M), N-terminal acetylation, and lysine acetylation. False discovery rates were set to 1% at both the protein and PSM levels.

### 2.12 Statistical analysis

Data were analyzed using SPSS 24.0. Normality and homogeneity of variance were assessed. For normally distributed data, one-way ANOVA was performed followed by either LSD test or Dunnett’s T3 test. Non-normally distributed data were analyzed using non-parametric tests. The significance level was set at *α* = 0.05, with *p* < 0.05 considered statistically significant.

## 3 Results

### 3.1 Electroacupuncture ameliorates motor function and increases the number of brain TH-positive neurons in PD mice induced by PD-FMT and MPTP

**Figure 1:**
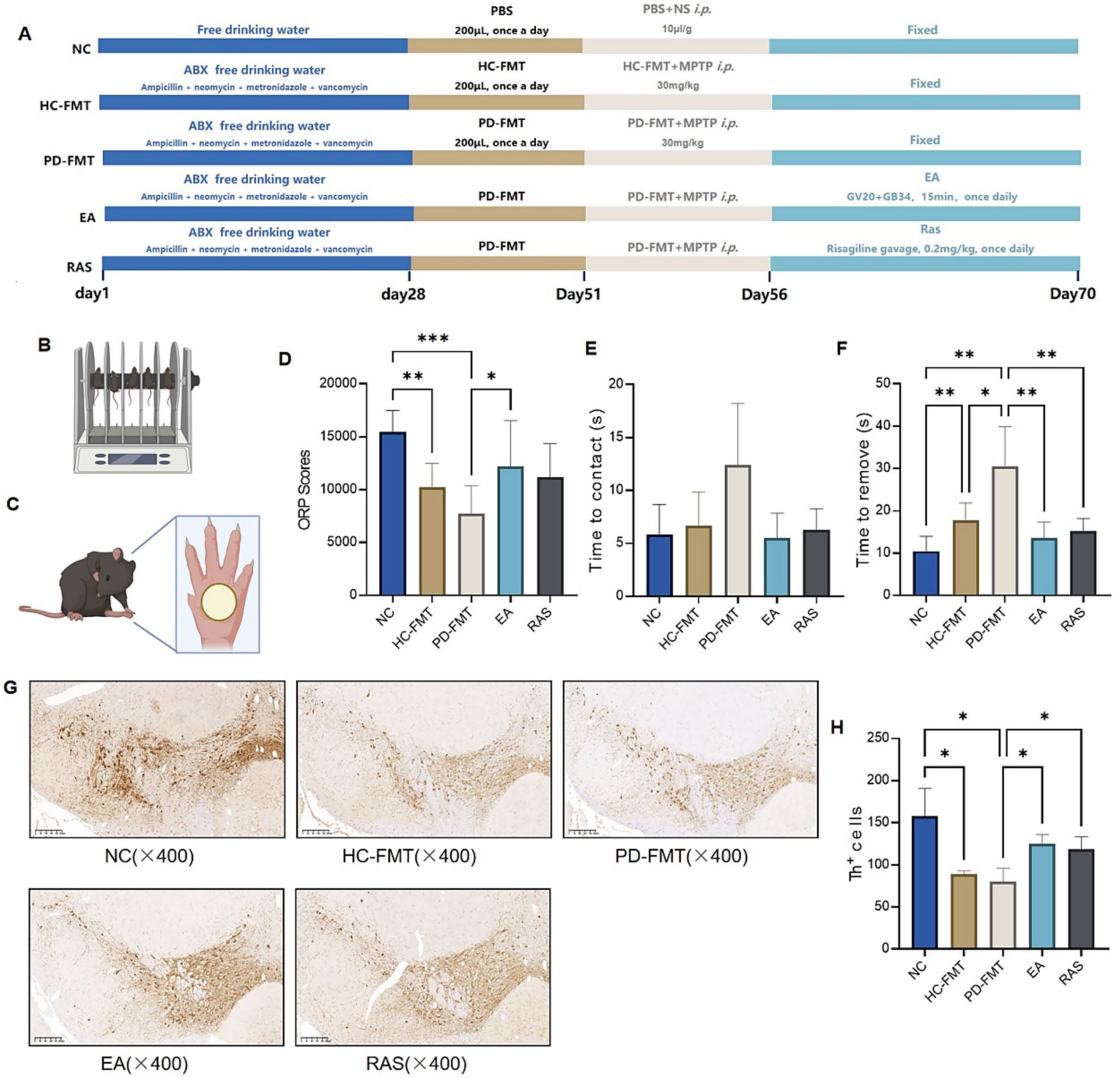
Electroacupuncture ameliorates motor function and increases the number of brain TH-positive neurons in PD mice induced by PD-FMT and MPTP. **(A)** Schematic diagram of the experimental procedure. **(B)** Schematic representation of the Rotarod test for mice. **(C)** Schematic representation of the remove adhesive dot test for mice. **(D)** Comparison of ORP (overall rating performance) scores among groups. **(E)** Comparison of time to contact the adhesive dot among groups. **(F)** Comparison of time to remove the adhesive dot among groups. **(G)** Representative images of TH^+^ neurons in the SNpc region via IHC staining (scale bar: 200 μm). **(H)** Quantification of TH^+^ neurons in the SNpc region across groups. NC, Normal control group; HC-FMT, Healthy control fecal microbiota transplantation group; PD-FMT, Parkinson’s disease fecal microbiota transplantation group; EA, Electroacupuncture group; RAS, Rasagiline group. *p < 0.05, **p < 0.01, ***p < 0.001. Behavioral assessments were performed in the following group sizes: NC, *n* = 10; HC-FMT, PD-FMT, EA, RAS, *n* = 9. For immunohistochemical analysis, the number of animals per group was: NC, *n* = 6; HC-FMT, PD-FMT, EA, RAS, *n* = 5.

To confirm the pathogenic effect of MPTP combined with PD-FMT and evaluate the therapeutic role of electroacupuncture, we systematically detected motor function and nigral dopaminergic neuron integrity in model mice. Rasagiline (RAS) was used as a positive control, while HC-FMT served as a donor-matched reference group to distinguish the specific effects of PD-derived microbiota (Figure 1A).

Behavioral and molecular analyses showed that MPTP combined with PD-FMT caused significant motor dysfunction and nigral dopaminergic neuron damage in mice. For gross motor function, the ORP score—an indicator of motor coordination—was significantly lower in both HC-FMT (*p* < 0.01) and PD-FMT groups (*p* < 0.001) than in the NC group. The PD-FMT group had a more obvious decrease (Figures 1B,D). Compared with the NC group, the PD-FMT group showed a trend toward longer time to contact the adhesive dot, but this difference was not statistically significant (Figure 1E). In contrast, the time to remove the adhesive dot was significantly prolonged in the PD-FMT group relative to the NC group (*p* < 0.01) (Figure 1F), which further confirmed fine motor impairment in PD model mice. At the molecular level, TH-positive neurons was significantly downregulated in the substantia nigra of both HC-FMT and PD-FMT groups compared with the NC group (*p* < 0.05) (Figures 1G,H). This indicated that MPTP combined with FMT (even with healthy donor microbiota) could induce dopaminergic neuron loss, and PD patient-derived microbiota worsened this damage.

Notably, EA effectively reversed these pathological changes in PD-FMT mice. For gross motor function, EA significantly increased the ORP score in the PD-FMT group(*p* < 0.05), while the RAS group only showed a non-significant upward trend in ORP scores (*p* > 0.05) relative to the PD-FMT group (Figures 1B,D). For fine motor function, both EA and RAS groups significantly shortened the adhesive dot removal time in PD-FMT mice (*p* < 0.01) (Figure 1F). For nigral dopaminergic neuron protection, EA significantly upregulated TH expression in the substantia nigra of PD-FMT mice (*p* < 0.05). This restorative effect was comparable to that of the positive control RAS (*p* < 0.05), confirming that EA could effectively preserve the number and function of nigral TH + neurons (Figures 1G,H).

### 3.2 Ywhaq hyperacetylation in PD nigra is attenuated by electroacupuncture

**Figure 2:**
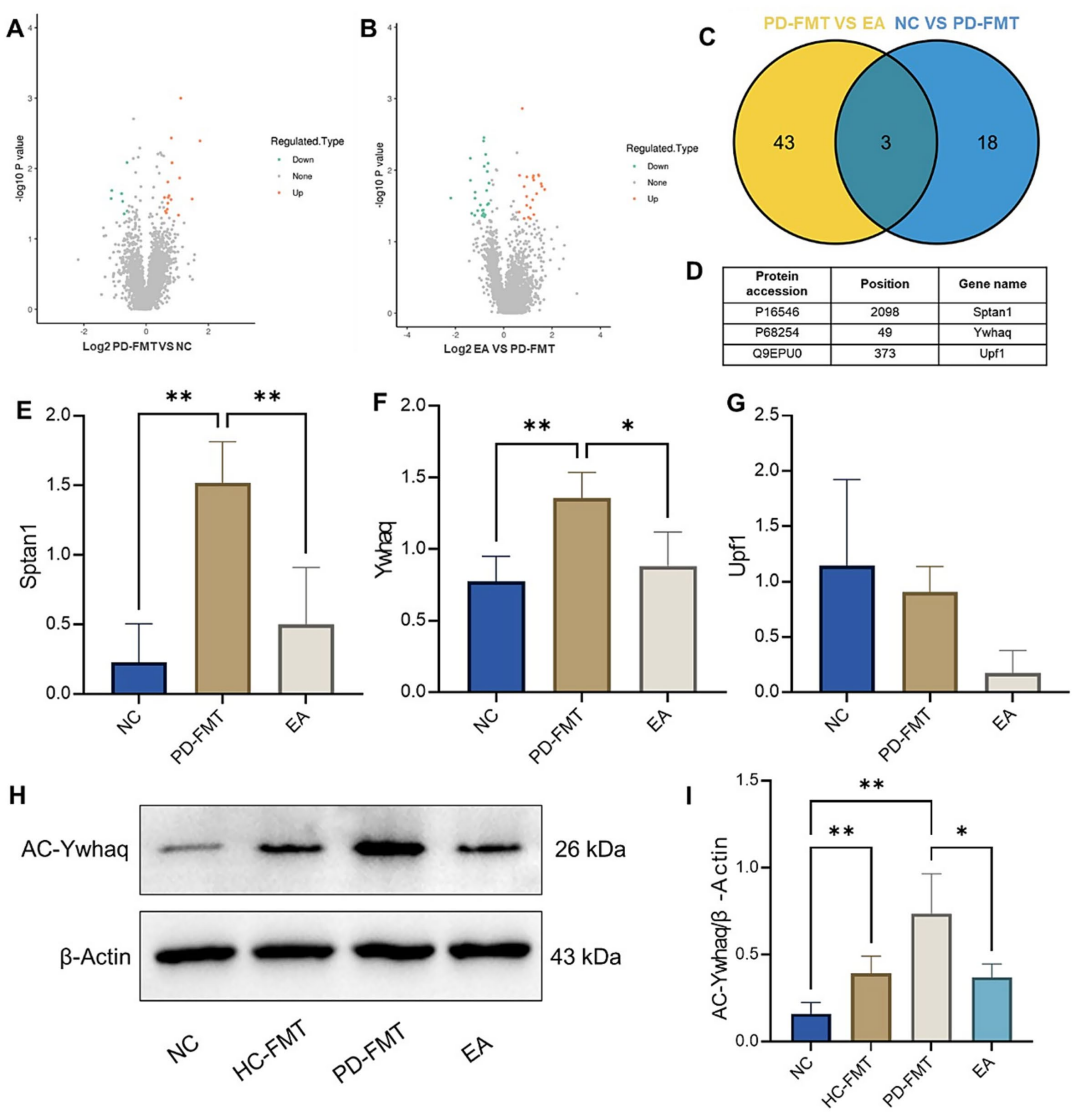
Effects of electroacupuncture on protein acetylation in the substantia nigra of PD mice. **(A)** Volcano plot: PD-FMT vs. NC. **(B)** Volcano plot: EA vs. PD-FMT. **(C)** Venn diagram of overlapping DAPs. **(D)** Heatmap of intersecting acetylated proteins. **(E–G)** Acetylation levels of Sptan1/Ywhaq/Upf1. **(H)** Representative WB bands for acetyl-Ywhaq. **(I)** Quantification of acetyl-Ywhaq/*β*-actin ratio. ^*^p < 0.05, **p < 0.01, *n* = 4.

To identify potential targets of electroacupuncture intervention in a PD mouse model induced by fecal microbiota transplantation from Parkinson’s disease patients, we performed 4D label-free acetylproteomic analysis on brain tissue proteins from NC, PD-FMT, and EA-treated mice. Data processing included acetylated peptide identification, reproducibility evaluation, quality control, screening of differentially acetylated proteins, along with Gene Ontology, Clusters of Orthologous Groups COG, and KEGG pathway enrichment analyses. The complete acetylproteomic dataset is provided in the Additional File 2. Differentially acetylated sites between groups were identified based on a fold change (FC) threshold of >1.5 for upregulation and <1/1.5 for downregulation, with a significance level of *p* < 0.05. Volcano plots revealed 16 significantly upregulated and 7 downregulated acetylation sites (corresponding to 16 and 7 proteins, respectively) in the PD-FMT group compared to the NC group (Figure 2A). In the EA group versus the PD-FMT group, 22 sites were upregulated and 27 downregulated (affecting 22 and 26 proteins, respectively) (Figure 2B). Intersection analysis of differentially acetylated proteins from the NC vs. PD-FMT and PD-FMT vs. EA comparisons identified three overlapping targets: Sptan1, Ywhaq, and Upf1 (Figures 2C,D). Acetylation levels of Sptan1 and Ywhaq were significantly increased in the substantia nigra of PD-FMT mice compared to NC controls (both *p* < 0.01), and EA treatment significantly reduced their acetylation relative to the PD-FMT group (*p* < 0.01 and *p* < 0.05) (Figures 2E–G). Based on literature evidence, Ywhaq was selected for further validation by Western blot. Consistent with the proteomic results, Ywhaq acetylation was significantly elevated in both the HC-FMT and PD-FMT groups compared to the NC group (*p* < 0.01), and EA treatment significantly decreased its acetylation compared to the PD-FMT group (*p* < 0.05) (Figures 2H,I).

### 3.3 Electroacupuncture improves open field behavior and reduces nigral *α*-synuclein accumulation in PD model mice

**Figure 3:**
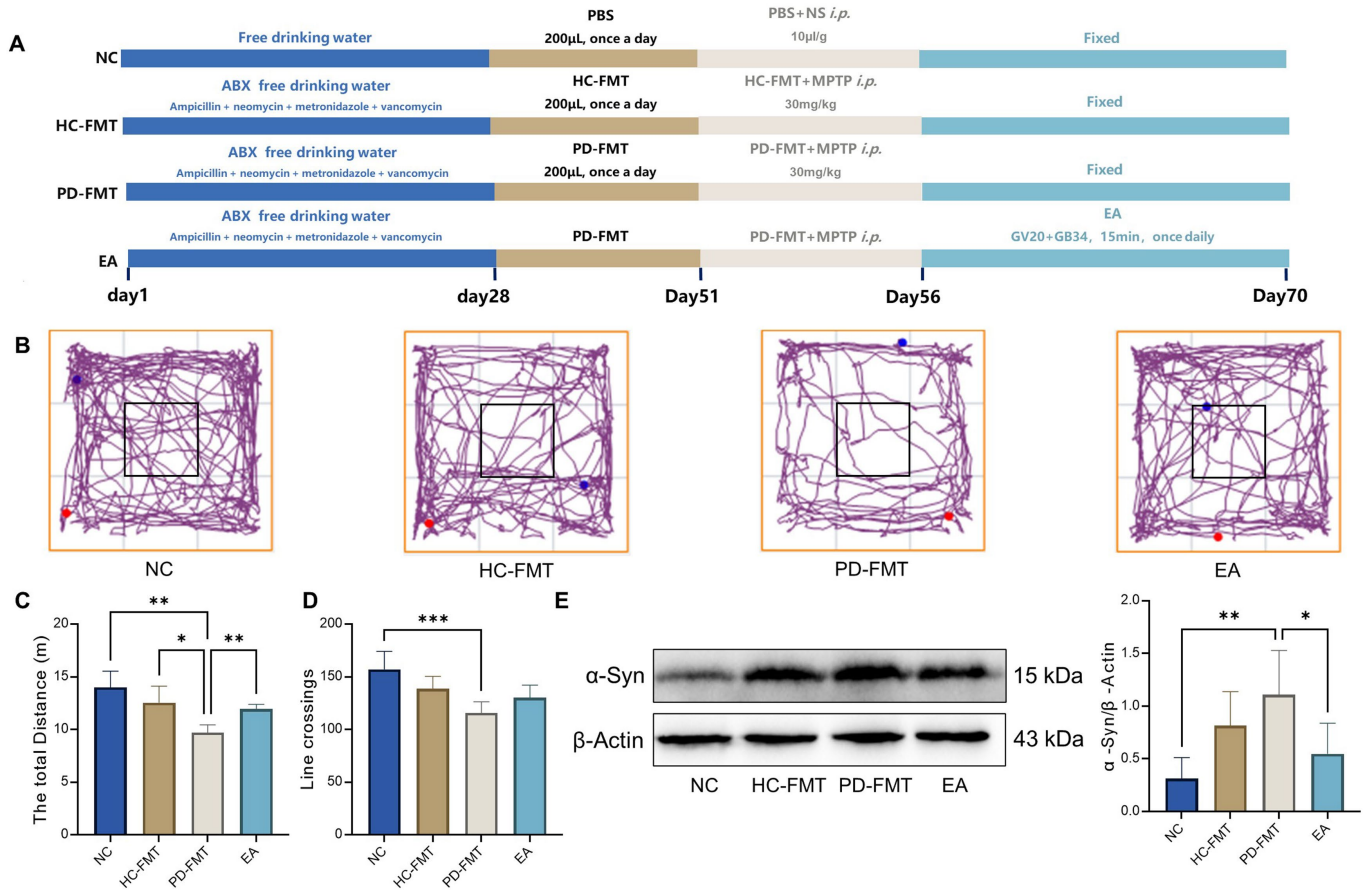
Electroacupuncture improves open field behavior and protects dopaminergic neurons in PD model mice. **(A)** Schematic diagram of the experimental procedure. **(B)** Representative locomotor tracking traces of mice in the open field test. Red dots indicate the starting points, and blue dots indicate the end points of movement. **(C)** Comparison of the total travel distance among groups. **(D)** Comparison of line-crossing. **(E)** Comparison of *α*-synuclein protein expression levels in the SNpc region across groups. NC, Normal control group; HC-FMT, Healthy control fecal microbiota transplantation group; PD-FMT, Parkinson’s disease fecal microbiota transplantation group; EA, Electroacupuncture group. ^*^p < 0.05, **p < 0.01, ***p < 0.001, *n* = 6.

Based on previous findings that EA modulates Ywhaq (14-3-3) acetylation in a PD mouse model, a second experimental batch was conducted to further investigate the regulatory effect of EA on histone deacetylases in the substantia nigra (Figure 3A). We also assessed EA’s influence on motor behavior using the open field test and evaluated *α*-synuclein accumulation via Western blot. The modeling and EA protocols remained consistent with the first batch. In the open field test, the PD-FMT group exhibited significantly reduced total movement distance (*p* < 0.01) and fewer line crossings (*p* < 0.001) compared to the NC group, indicating impaired locomotor and exploratory behavior. Relative to the PD-FMT group, the HC-FMT group showed a moderate increase in total distance (*p* < 0.05), while the EA group demonstrated a more substantial improvement (*p* < 0.001) (Figures 3B–D). Western blot analysis revealed a significant upregulation of α-synuclein protein in the substantia nigra of PD-FMT mice compared to the NC group (*p* < 0.01), confirming that MPTP combined with PD-FMT promotes *α*-synuclein aggregation. Notably, EA treatment markedly reduced *α*-synuclein expression relative to the PD-FMT group (*p* < 0.05), supporting its role in mitigating pathological protein accumulation in PD mice (Figure 3E).

### 3.4 Electroacupuncture reverses reduced expression of acetylation-modifying enzymes in the substantia nigra of PD model mice

HDAC1, HDAC2, HDAC3, SIRT1, and SIRT2 are key deacetylases involved in the removal of acetyl groups from proteins (Manjula et al., 2020; Moreno-Yruela et al., 2022). To investigate their expression in Parkinson’s disease, we assessed both protein and mRNA levels of these enzymes in the substantia nigra across experimental groups using Western blot and quantitative real-time PCR.

**Figure 4:**
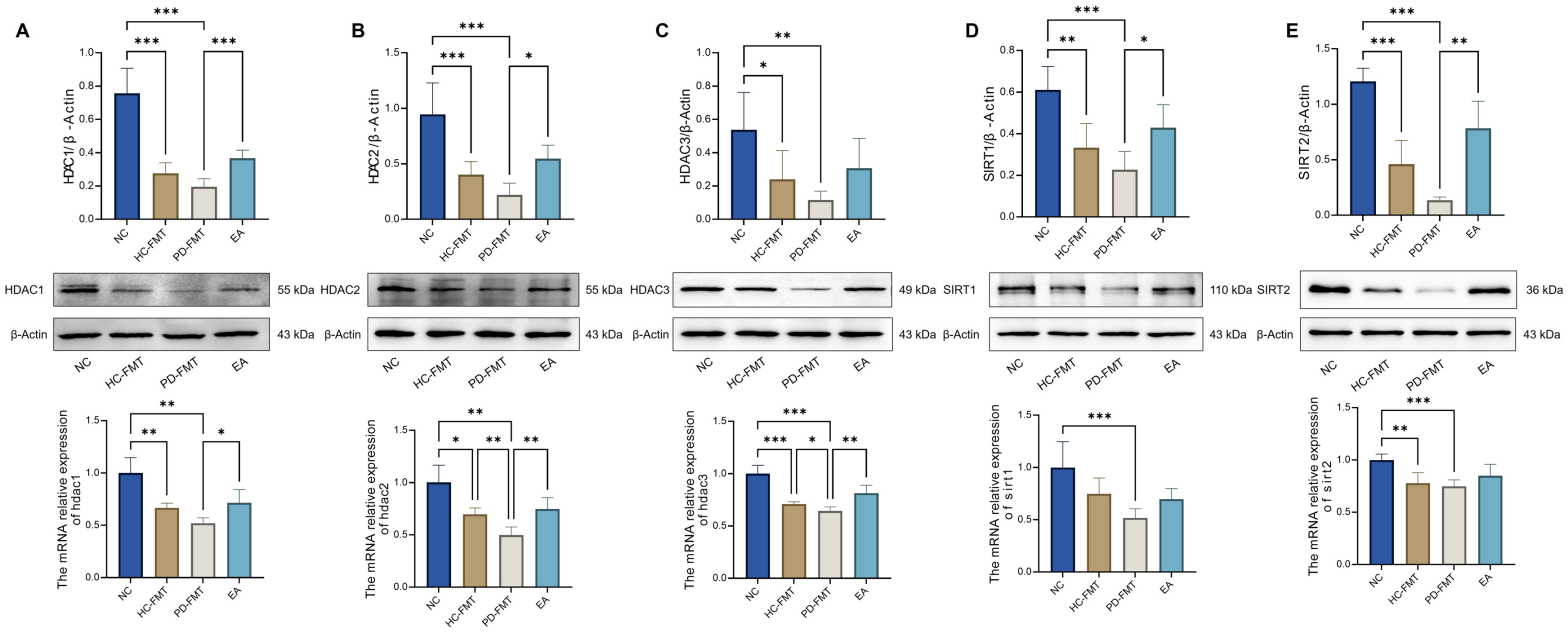
Effects of electroacupuncture on protein and mRNA expression of acetylation-modifying enzymes in the substantia nigra of PD mice. **(A)** HDAC1 protein and mRNA expression comparison. **(B)** HDAC2 protein and mRNA expression comparison. **(C)** HDAC3 protein and mRNA expression comparison. **(D)** SIRT1 protein and mRNA expression comparison. **(E)** SIRT2 protein and mRNA expression comparison. NC, Normal control group; HC-FMT, healthy control fecal microbiota transplantation group; PD-FMT, Parkinson’s disease fecal microbiota transplantation group; EA, electroacupuncture group. ^*^p < 0.05, ^**^p < 0.01, ^***^p < 0.001, *n = 6.*

Compared with the NC group, both the PD-FMT and HC-FMT groups exhibited significantly reduced protein and mRNA expression of HDAC1, HDAC2, HDAC3, and SIRT2 (*p* < 0.05). Additionally, the PD-FMT group showed a marked decrease in SIRT1 expression at both protein and mRNA levels relative to the NC group (*p* < 0.05). Notably, EA treatment significantly counteracted these alterations: compared to the PD-FMT group, the EA group demonstrated increased protein expression of HDAC1, HDAC2, SIRT1, and SIRT2 (*p* < 0.05), and elevated mRNA levels of HDAC1, HDAC2, and HDAC3 (*p* < 0.05) (Figures 4A–E). These findings suggest that EA can effectively restore the expression of major deacetylases involved in PD-related protein acetylation dysregulation.

## 4 Discussion

Parkinson’s disease is a progressive neurodegenerative disorder characterized by selective loss of dopaminergic neurons in the substantia nigra pars compacta and pathological accumulation of misfolded *α*-synuclein in Lewy bodies and Lewy neurites (Wakabayashi et al., 2013). Clinical manifestations include classic motor symptoms—resting tremor, bradykinesia, rigidity, and postural instability—as well as non-motor features such as gastrointestinal dysfunction, olfactory impairment, and neuropsychiatric disturbances, which often precede motor deficits by years and underscore the systemic nature of the disease (Carabotti et al., 2015; Shannon et al., 2012; Tanner and Ostrem, 2024).

The etiology of PD involves complex genetic and environmental interactions. Mutations in genes such as LRRK2, Parkin, and PINK1 disrupt mitochondrial function, protein clearance, and neuroinflammatory responses. Environmental factors—including neurotoxin exposure, head trauma, and chronic gut inflammation—further increase susceptibility. Recently, gut microbiota dysregulation (Hirayama and Ohno, 2021; Yemula et al., 2021; Zhang X. et al., 2023) and impaired protein acetylation homeostasis (Wang R. et al., 2020; Yakhine-Diop et al., 2019; Esteves et al., 2019) have emerged as key mechanisms in PD pathogenesis.

Our previous clinical studies demonstrated that acupuncture improves motor function in PD patients, as reflected by increased step length, reduced UPDRS scores, and shorter walking time (Li et al., 2020). Preclinical studies showed that acupuncture reduces *α*-synuclein accumulation, enhances lysosomal function, and protects dopaminergic neurons in MPTP-induced PD mice (Tian et al., 2016). However, acupuncture could significantly regulate lysosomal function, clear accumulated *α*-synuclein in the brains of PD mice, protect neurons, and improve abnormal behavioral performances in PD mice. The present study reproduced these previous findings. Another study revealed that acupuncture could protect dopaminergic neurons in PD mice, improve their motor function (Gao et al., 2022), and enhance gut microbial diversity as well as regulate gut microbiota abundance, confirming that the regulatory effect of acupuncture on gut microbiota may be one of the key mechanisms underlying its neuroprotective effects (Wang Z. Q. et al., 2020). These findings motivated the current investigation into the mechanisms of electroacupuncture (EA), particularly through gut-microbiota and protein-acetylation pathways.

In this study, electroacupuncture exerted therapeutic effects on PD model mice induced by MPTP combined with PD patient fecal microbiota transplantation. Behavioral assessments showed EA significantly ameliorated motor deficits in PD-FMT mice—improving ORP scores, shortening the adhesive dot removal time, and increasing open-field total distance—reversing the impaired performance relative to normal controls. At the molecular level, EA restored reduced substantia nigra tyrosine hydroxylase expression and decreased elevated *α*-synuclein accumulation in PD-FMT mice. Using 4D label-free acetylproteomics, we identified hyperacetylation of Sptan1 and Ywhaq (14-3-3) in PD-FMT mice, which was reversed by EA. We further validated that EA reduced Ywhaq acetylation and restored the expression of deacetylases (HDAC1/2/3, SIRT1/2) at both protein and mRNA levels. These results suggest that EA mitigates PD-related hyperacetylation at least partly by upregulating deacetylase expression.

Gut microbiota dysbiosis has been identified as an early and critical driver of PD pathogenesis (Claudino Dos Santos et al., 2023; Zhang W. et al., 2023). Clinical studies consistently demonstrate that PD patients exhibit a distinct gut microbial signature, characterized by reduced abundance of short-chain fatty acid (SCFA)-producing bacteria including *Faecalibacterium* and *Roseburia*, and increased levels of pro-inflammatory taxa such as *Akkermansia* and *Escherichia* (Wang R. et al., 2020; Unger et al., 2016; Nishiwaki et al., 2020). This dysbiosis compromises intestinal barrier integrity, evidenced by downregulated expression of tight junction proteins including occludin and ZO-1 (Ioannou et al., 2024; Liao et al., 2024; Clairembault et al., 2015), enabling translocation of microbial metabolites such as lipopolysaccharides and SCFAs into the systemic circulation. Elevated peripheral SCFAs, in particular, correlate with PD severity, potentially via inhibiting HDACs and promoting microglial activation (Chen et al., 2022). Transplanting PD patient microbiota into mice recapitulates motor deficits and *α*-synuclein pathology (Sampson et al., 2016), while fecal microbiota transplantation from healthy donors may mildly improve motor symptoms in early PD (Bruggeman et al., 2024). To recapitulate this “gut-brain axis” dysfunction in preclinical models, our study employed a dual modeling approach: First, PD patient fecal microbiota transplantation induced gut microbial dysregulation in mice, followed by MPTP administration to trigger dopaminergic neuron loss. This approach mimics both intestinal and central pathological features of PD, providing a model to investigate cross-talk between gut dysbiosis and neurodegeneration (Sampson et al., 2016; Sun et al., 2018; Xie et al., 2023). In our study, electroacupuncture further showed the ability to alleviate pathological effects induced by gut microbiota dysregulation in PD-FMT mice. This may indirectly support its potential to mitigate gut-brain axis-related impairment, thereby reducing subsequent damage to the central nervous system. Although the current study did not directly assess gut-derived mediators, EA’s efficacy in this model suggests a potential role in modulating gut-brain communication, a hypothesis warranting further investigation.

Lysine acetylation is a reversible post-translational modification regulated by acetyltransferases and deacetylases (KDACs), including Zn^2+^-dependent HDACs and NAD^+^-dependent sirtuins (SIRTs) (Hosp et al., 2017). Acetylation modulates diverse cellular processes—transcription, signaling, protein aggregation, autophagy—and its dysregulation is implicated in neurodegeneration (Narita et al., 2019). PD patients and models show downregulation of HDAC1/2 and SIRT1 (Yakhine-Diop et al., 2019; Park et al., 2016), leading to hyperacetylation of non-histone proteins.

We found that hyperacetylation of Ywhaq (14-3-3) at Lys49—a residue located within its functional binding groove—is associated with PD-like pathology and reversed by EA. Acetylation neutralizes the positive charge of lysine, potentially impairing 14-3-3’s ability to bind client proteins (Mortenson et al., 2015). 14-3-3 proteins are highly expressed in the brain and play key roles in stabilizing phosphorylated proteins, regulating subcellular localization, and preventing aggregation (Xu et al., 2013; Karlberg et al., 2018). They interact with multiple PD-related proteins, including LRRK2, *α*-synuclein, and parkin. For example, 14-3-3 binding maintains LRRK2 phosphorylation and activity (Manschwetus et al., 2020), while 14-3-3θ/*τ* inhibits α-synuclein aggregation and promotes refolding (Plotegher et al., 2014; Giusto et al., 2021). Reduced 14-3-3 function exacerbates neurotoxicity, whereas overexpression is neuroprotective (Yacoubian et al., 2010; Cho et al., 2023). Thus, acetylation-mediated disruption of 14–3-3 function could contribute to PD pathogenesis, and deacetylation by EA may restore its neuroprotective role.

Several limitations should be noted. The acute MPTP+PD-FMT model does not fully recapitulate chronic, progressive PD in humans. The downstream effects of 14-3-3 acetylation—particularly its interaction with PD-related proteins—remain unclear, and direct evidence linking EA to gut-brain axis mediators (e.g., SCFAs) is lacking. Future studies should employ chronic models, investigate 14–3-3 client protein networks, and quantify microbial metabolites.

## 5 Conclusion

In conclusion, EA improves behavioral and neuropathological phenotypes in a PD mouse model by restoring deacetylase expression and normalizing protein acetylation, particularly of 14-3-3. Our results underscore the therapeutic potential of EA and highlight acetylation modulation as a promising strategy for PD treatment.

## Data Availability

The datasets presented in this study can be found in online repositories. The names of the repository/repositories and accession number(s) can be found in the article/Supplementary material.
