# Supplementary material for: Electroacupuncture alleviates Parkinson’s disease by targeting HDAC/SIRT-mediated deacetylation of 14-3-3
Source: Front Aging Neurosci. 2026 Jan 14;17:1719326. doi: 10.3389/fnagi.2025.1719326 (PMC12847356; doi:10.3389/fnagi.2025.1719326)
Supplement: Supplementary file 1 [file Supplementary_file_1.docx]

**Methods of 4D label-free Acetylproteomic Analysis**

**Protein Extraction**
Samples were retrieved from -80°C storage. An appropriate amount of tissue was weighed into a liquid nitrogen-cooled mortar, fully ground to powder under liquid nitrogen. Four volumes of lysis buffer (8 M urea, 1% protease inhibitor cocktail, 3 μM TSA, 50 mM NAM) were added to the powdered tissue per sample group, followed by sonication. After centrifugation at 12,000 g for 10 min at 4°C to remove debris, the supernatant was transferred to a new tube. Protein concentration was determined using a BCA assay kit.

**Trypsin Digestion**
Equal protein amounts from each sample were digested. Volumes were adjusted to uniformity using lysis buffer. Trichloroacetic acid (TCA) was slowly added to a final concentration of 20%, vortexed, and precipitated at 4°C for 2 h. After centrifugation at 4,500 g for 5 min, the supernatant was discarded. The pellet was washed 2–3 times with ice-cold acetone, air-dried, and resuspended in 200 mM TEAB via sonication. Trypsin was added at a 1:50 (w/w) enzyme-to-protein ratio for overnight digestion. Dithiothreitol (DTT) was added to 5 mM final concentration and incubated at 56°C for 30 min for reduction. Subsequently, iodoacetamide (IAA) was added to 11 mM final concentration and incubated at room temperature for 15 min in the dark.

**Enrichment of Post-Translational Modifications**
Peptides were dissolved in IP buffer (100 mM NaCl, 1 mM EDTA, 50 mM Tris-HCl, 0.5% NP-40, pH 8.0). The supernatant was transferred to pre-washed acetylated antibody beads (PTM-104, PTM Bio, Hangzhou Jingjie Biotechnology Co., Ltd.). The mixture was incubated on a rotary shaker at 4°C overnight with gentle agitation. Beads were sequentially washed four times with IP buffer and twice with deionized water. Peptides were eluted three times using 0.1% trifluoroacetic acid (TFA). The eluate was collected and lyophilized. Desalting was performed using C18 ZipTips according to the manufacturer’s instructions. The desalted peptides were lyophilized for LC-MS/MS analysis.

**Liquid Chromatography-Tandem Mass Spectrometry (LC-MS/MS) Analysis**
Peptides were dissolved in mobile phase A (0.1% formic acid, 2% acetonitrile in water) and separated using a NanoElute ultra-high-performance liquid chromatography (UHPLC) system. Mobile phase B consisted of 0.1% formic acid in 100% acetonitrile. The gradient program was: 0–44 min, 6%–22% B; 44–54 min, 22%–30% B; 54–57 min, 30%–80% B; 57–60 min, 80% B (flow rate: 450 nL/min). Eluted peptides were ionized in a Capillary ion source (1.7 kV) and analyzed by a timsTOF Pro mass spectrometer. Both precursor and fragment ions were detected using high-resolution TOF. MS/MS scanning range:*m/z*100–1700. Data acquisition employed Parallel Accumulation Serial Fragmentation (PASEF) mode. After each full MS scan (mass range: 100–1700 *m/z*), 10 PASEF MS/MS scans were performed for precursors with charge states 0–5. Dynamic exclusion was set to 30 s.

**Database Search**
Raw MS data were processed using MaxQuant (v1.6.15.0). Search parameters: Database: Mus_musculus_10090_SP_20210721.fasta (17,089 entries) with reverse decoy sequences for FDR calculation and common contaminants. Enzyme specificity: Trypsin/P; Max missed cleavages: 4; Min peptide length: 7 amino acids; Max modifications per peptide: 5; Precursor mass tolerance: 20 ppm (first search and main search); Fragment mass tolerance: 20 ppm. Fixed modification: Carbamidomethyl (C); Variable modifications: Methionine oxidation, N-terminal acetylation, Lysine acetylation. Protein and PSM false discovery rates (FDR) were set to 1%.

**Bioinformatics Methods**

**Annotation Methods**

GO Annotation: The Gene Ontology, or GO, is a major bioinformatics initiative to unify the representation of gene and gene product attributes across all species. More specifically, the project aims to: 1. Maintain and develop its controlled vocabulary of gene and gene product attributes; 2. Annotate genes and gene products, and assimilate and disseminate annotation data; 3. Provide tools for easy access to all aspects of the data provided by the project. The ontology covers three domains: 1. Cellular component: A cellular component is just that, a component of a cell, but with the proviso that it is part of some larger object; this may be an anatomical structure (e.g. rough endoplasmic reticulum or nucleus) or a gene product group (e.g. ribosome, proteasome or a protein dimer). 2. Molecular function: Molecular function describes activities, such as catalytic or binding activities, that occur at the molecular level. GO molecular function terms represent activities rather than the entities (molecules or complexes) that perform the actions, and do not specify where or when, or in what context, the action takes place. 3. Biological process: A biological process is series of events accomplished by one or more ordered assemblies of molecular functions. It can be difficult to distinguish between a biological process and a molecular function, but the general rule is that a process must have more than one distinct steps. Gene Ontology (GO) annotation proteome was derived from the UniProt-GOA database ( <http://www.ebi.ac.uk/GOA/>). Firstly, Converting identified protein ID to UniProt ID and then mapping to GO IDs by protein ID. If some identified proteins were not annotated by UniProt-GOA database, the InterProScan soft would be used to annotated protein’s GO functional based on protein sequence alignment method. Then proteins were classified by Gene Ontology annotation based on three categories: biological process, cellular component and molecular function.

Domain Annotation: A protein domain is a conserved part of a given protein sequence and structure that can evolve, function and exist independently of the rest of the protein chain. Each domain forms a compact three-dimensional structure and often can be independently stable and folded. Many proteins consist of several structural domains. One domain may appear in a variety of differentially expressed proteins. Molecular evolution uses domains as building blocks and these may be recombined in different arrangements to create proteins with different functions. Domains vary in length from between about 25 amino acids up to 500 amino acids in length. The shortest domains such as zinc fingers are stabilized by metal ions or disulfide bridges. Domains often form functional units, such as the calcium-binding EF hand domain of calmodulin. Because they are independently stable, domains can be “swapped” by genetic engineering between one protein and another to make chimeric proteins.Identified proteins domain functional description were annotated by InterProScan (a sequence analysis application) based on protein sequence alignment method, and the InterPro domain database was used. InterPro (<http://www.ebi.ac.uk/interpro/>) is a database that integrates diverse information about protein families, domains and functional sites, and makes it freely available to the public via Web-based interfaces and services. Central to the database are diagnostic models, known as signatures, against which protein sequences can be searched to determine their potential function. InterPro has utility in the large-scale analysis of whole genomes and meta-genomes, as well as in characterizing individual protein sequences.

KEGG Pathway Annotation: KEGG connects known information on molecular interaction networks, such as pathways and complexes (the “Pathway” database), information about genes and proteins generated by genome projects (including the gene database) and information about biochemical compounds and reactions (including compound and reaction databases). These databases are different networks, known as the “protein network”, and the “chemical universe” respectively. There are efforts in progress to add to the knowledge of KEGG, including information regarding ortholog clusters in the KEGG Orthology database. KEGG Pathways mainly including: Metabolism, Genetic Information Processing, Environmental Information Processing, Cellular Processes, Rat Diseases, Drug development. Kyoto Encyclopedia of Genes and Genomes (KEGG) database was used to annotate protein pathway. Firstly, using KEGG online service tools KAAS to annotated protein’s KEGG database description. Then mapping the annotation result on the KEGG pathway database using KEGG online service tools KEGG mapper.

Subcellular Localization: The cells of eukaryotic organisms are elaborately subdivided into functionally distinct membrane bound compartments. Some major constituents of eukaryotic cells are: extracellular space, cytoplasm, nucleus, mitochondria, Golgi apparatus, endoplasmic reticulum (ER), peroxisome, vacuoles, cytoskeleton, nucleoplasm, nucleolus, nuclear matrix and ribosomes. Bacteria also have subcellular localizations that can be separated when the cell is fractionated. The most common localizations referred to include the cytoplasm, the cytoplasmic membrane (also referred to as the inner membrane in Gram-negative bacteria), the cell wall (which is usually thicker in Gram-positive bacteria) and the extracellular environment. Most Gram-negative bacteria also contain an outer membrane and periplasmic space. Unlike eukaryotes, most bacteria contain no membrane-bound organelles, however there are some exceptions. There, we used wolfpsort a subcellular localization predication soft to predict subcellular localization. Wolfpsort is an updated version of PSORT/PSORT II for the prediction of eukaryotic sequences. Special for protokaryon species, Subcellular localization prediction soft CELLO was used.

**Functional Enrichment**

Enrichment of Gene Ontology analysis: Proteins were classified by GO annotation into three categories: biological process, cellular compartment and molecular function. For each category, a two-tailed Fisher’s exact test was employed to test the enrichment of the differentially expressed protein against all identified proteins. The GO with a corrected p-value < 0.05 is considered significant. Enrichment of pathway analysis: Encyclopedia of Genes and Genomes (KEGG) database was used to identify enriched pathways by a two-tailed Fisher’s exact test to test the enrichment of the differentially expressed protein against all identified proteins. The pathway with a corrected p-value < 0.05 was considered significant. These pathways were classified into hierarchical categories according to the KEGG website. Enrichment of protein domain analysis: For each category proteins, InterPro (a resource that provides functional analysis of protein sequences by classifying them into families and predicting the presence of domains and important sites) database was researched and a two-tailed Fisher’s exact test was employed to test the enrichment of the differentially expressed protein against all identified proteins. Protein domains with a corrected p-value < 0.05 were considered significant.

**Enrichment-based Clustering**

For further hierarchical clustering based on differentially expressed protein functional classification (such as: GO, Domain, Pathway, Complex). We first collated all the categories obtained after enrichment along with their P values, and then filtered for those categories which were at least enriched in one of the clusters with P value <0.05. This filtered P value matrix was transformed by the function x = −log10 (P value). Finally these x values were z-transformed for each functional category. These z scores were then clustered by one-way hierarchical clustering (Euclidean distance, average linkage clustering) in Genesis. Cluster membership were visualized by a heat map using the “heatmap.2” function from the “gplots” R-package.

**Protein-protein Interaction Network**

All differentially expressed protein database accession or sequence were searched against the STRING database version 11.0 for protein-protein interactions. Only interactions between the proteins belonging to the searched data set were selected, thereby excluding external candidates. STRING defines a metric called “confidence score” to define interaction confidence; we fetched all interactions that had a confidence score ≥ 0.7 (high confidence). Interaction network form STRING was visualized in R package “networkD3”.
