# Supplementary material for: Electroacupuncture alleviates Parkinson’s disease by targeting HDAC/SIRT-mediated deacetylation of 14-3-3
Source: Front Aging Neurosci. 2026 Jan 14;17:1719326. doi: 10.3389/fnagi.2025.1719326 (PMC12847356; doi:10.3389/fnagi.2025.1719326)
Supplement: Supplementary file 2 [file Supplementary_file_2.docx]

**Effects of Electroacupuncture on Protein Acetylation in the Substantia Nigra of PD Mice**

To identify potential targets of electroacupuncture intervention in a PD model established via fecal microbiota transplantation (FMT) from PD patients, we performed 4D label-free acetylproteomics on brain tissue proteins from three experimental groups:
(1) Normal control (NC)；(2) PD-FMT mice；(3) EA mice.

**Identification of Acetylated Peptides**

Tandem mass spectrometry data were analyzed using MaxQuant (v1.6.15.0) with the following parameters:

- Database: Mus_musculus_10090_SP_20210721.fasta (17,089 sequences)
- Decoy database: Included for false discovery rate (FDR) calculation
- Contaminant database: Added to filter out contaminant proteins
- Digestion enzyme: Trypsin/P (maximum 4 missed cleavages)
- Shortest peptide length: 7 amino acids
- Maximum modifications per peptide: 5
- Mass tolerance: 20 ppm (MS1 and MS2)
- Fixed modification: Carbamidomethylation (C)
- Variable modifications: Oxidation (M), N-terminal acetylation, Lysine acetylation
- FDR threshold: 1% (protein and PSM levels)

A total of 729,898 MS/MS spectra were acquired. After database searching, 112,689 spectra were matched, identifying 14,214 peptides and 8,166 acetylated peptides. These mapped to 2,952 proteins with 8,322 acetylation sites, of which 6,279 sites across 2,286 proteins were quantifiable (Fig. 4).


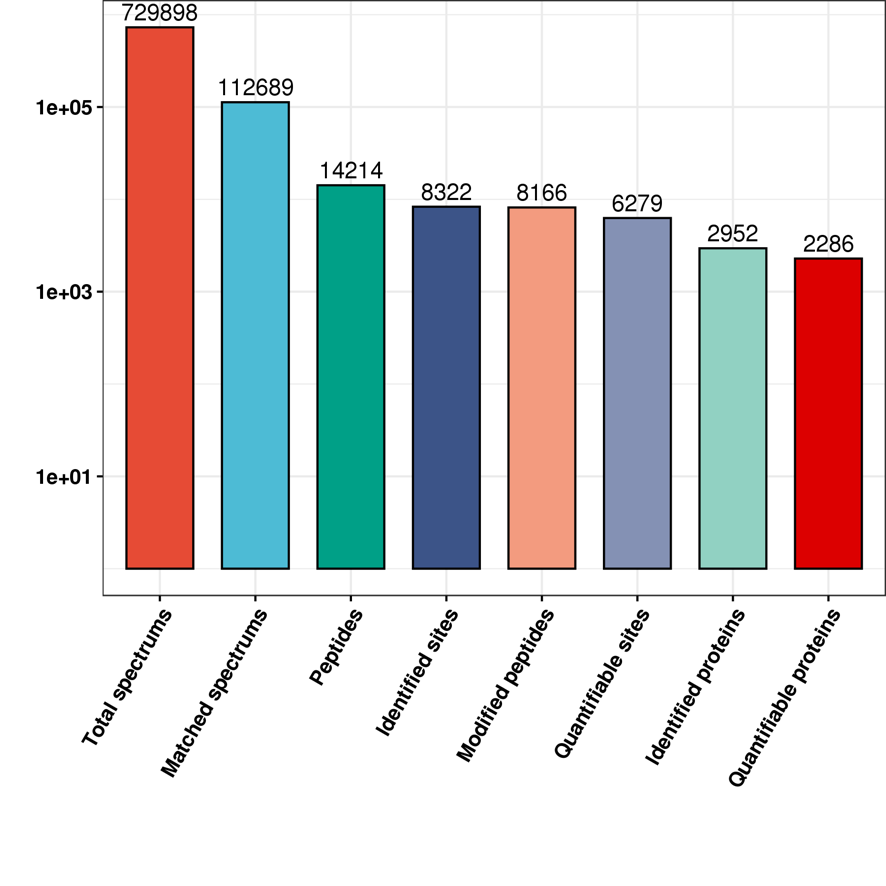


**Figure 5. Overview of Protein Identification**

**Quality Control and Reproducibility**

Peptide length distribution showed most of peptides were 7–20 residues long (Fig. 6A). For biological replicates (n=4/group):

- Relative standard deviation (RSD): <0.4 across all groups (Fig. 6B)
- Pearson’s correlation coefficient (PCC): around 1 between replicates (Fig. 6C)


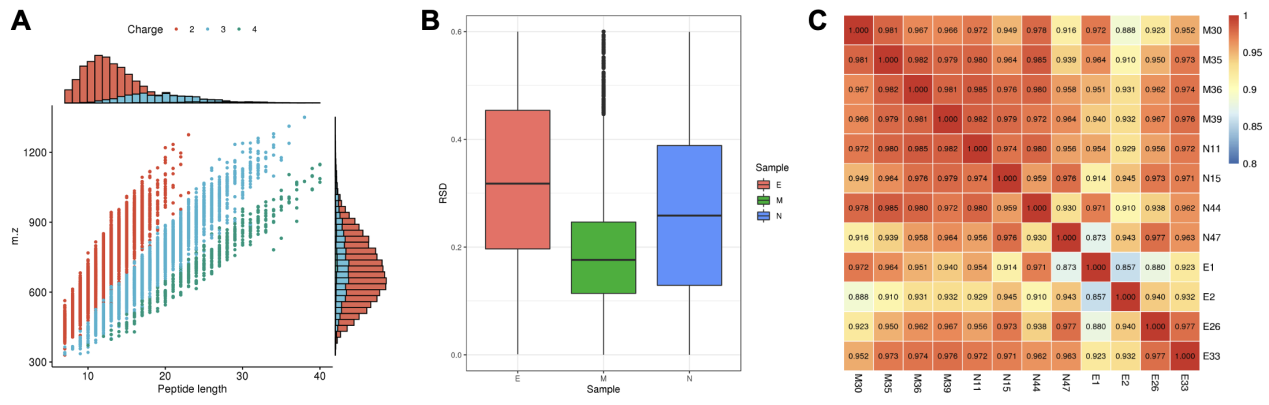


**Figure 6. Data Quality Assessment**

(A) Peptide length distribution; (B) RSD box plots; (C) PCC heatmap

**Screening of Differentially Acetylated Proteins**

Differentially acetylated proteins (DAPs) were defined by:

1. |Fold change (FC)| ≥1.5; (2) P-value <0.05

Volcano plots demonstrated:

- PD-FMT vs. NC: 16 upregulated sites (16 proteins); 7 downregulated sites (7 proteins) (Fig. 7, Table 2)
- EA vs. PD-FMT: 22 upregulated sites (22 proteins); 27 downregulated sites (26 proteins) (Fig. 8, Table 3)


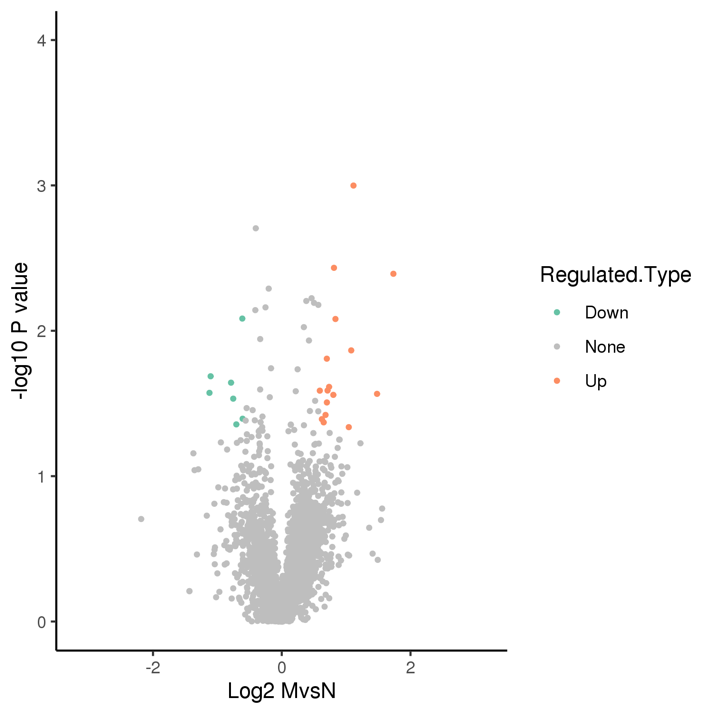


**Figure 7. Volcano plot:** **PD-FMT vs. NC**

**Table 2. Significantly Altered Acetylation Sites (PD-FMT vs. NC)**

| **Protein accession** | **Position** | **M/N Ratio** | **M/N P value** | **Regulated Type** | **Protein description** | **Gene name** |
| --- | --- | --- | --- | --- | --- | --- |
| P16546 | 2098 | 3.326 | 0.0040559 | Up | Spectrin alpha chain, non-erythrocytic 1 | Sptan1 |
| P46096 | 297 | 1.57 | 0.0426733 | Up | Synaptotagmin-1 | Syt1 |
| P49312 | 8 | 2.79 | 0.0271671 | Up | Heterogeneous nuclear ribonucleoprotein A1 | Hnrnpa1 |
| P49962 | 60 | 1.742 | 0.0276226 | Up | Signal recognition particle 9 kDa protein | Srp9 |
| P63101 | 49 | 1.625 | 0.01556 | Up | 14-3-3 protein zeta/delta | Ywhaz |
| P62748 | 174 | 1.602 | 0.0378998 | Up | Hippocalcin-like protein 1 | Hpcal1 |
| P68254 | 49 | 1.754 | 0.0036887 | Up | 14-3-3 protein theta | Ywhaq |
| Q64332 | 382 | 1.637 | 0.025734 | Up | Synapsin-2 | Syn2 |
| Q80XN0 | 103 | 1.626 | 0.0311297 | Up | D-beta-hydroxybutyrate dehydrogenase, mitochondrial | Bdh1 |
| Q8VCE6 | 141 | 2.164 | 0.0010028 | Up | 5'(3')-deoxyribonucleotidase, mitochondrial | Nt5m |
| Q91VR8 | 30 | 2.112 | 0.0136558 | Up | Protein BRICK1 | Brk1 |
| Q99KR7 | 189 | 1.54 | 0.0405625 | Up | Peptidyl-prolyl cis-trans isomerase F, mitochondrial | Ppif |
| Q9D8E6 | 181 | 1.664 | 0.0243246 | Up | 60S ribosomal protein L4 | Rpl4 |
| Q9QYR6 | 210 | 2.057 | 0.045976 | Up | Microtubule-associated protein 1A | Map1a |
| Q9R1T4 | 185 | 1.783 | 0.0082978 | Up | Septin-6 | Septin6 |
| Q9Z2I8 | 140 | 1.506 | 0.0258171 | Up | Succinate--CoA ligase [GDP-forming] subunit beta, mitochondrial | Suclg2 |
| B2RSH2 | 132 | 0.613 | 0.0440664 | Down | Guanine nucleotide-binding protein G(i) subunit alpha-1 | Gnai1 |
| Q01853 | 109 | 0.656 | 0.0402815 | Down | Transitional endoplasmic reticulum ATPase | Vcp |
| Q62425 | 77 | 0.654 | 0.0082326 | Down | Cytochrome c oxidase subunit NDUFA4 | Ndufa4 |
| Q8BGH4 | 101 | 0.579 | 0.0227524 | Down | Receptor expression-enhancing protein 1 | Reep1 |
| Q91V61 | 169 | 0.459 | 0.0267475 | Down | Sideroflexin-3 | Sfxn3 |
| Q9DBP5 | 88 | 0.465 | 0.0205452 | Down | UMP-CMP kinase | Cmpk1 |
| Q9EPU0 | 373 | 0.593 | 0.0293402 | Down | Regulator of nonsense transcripts 1 | Upf1 |


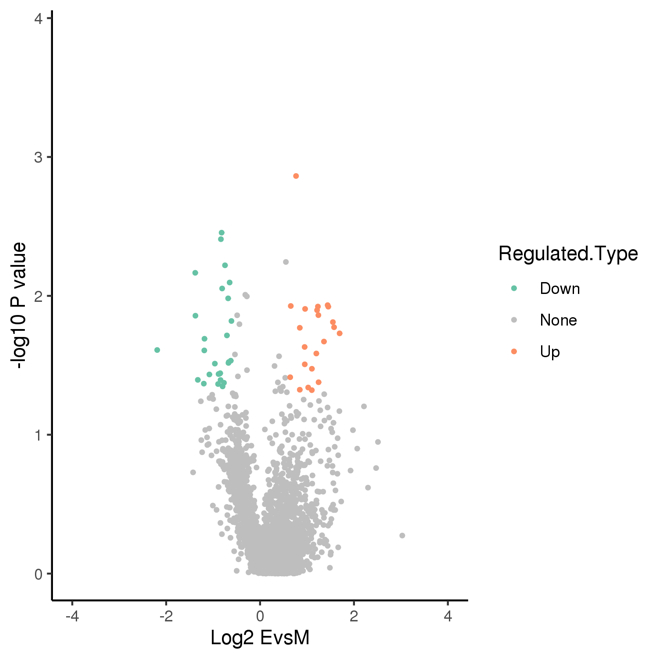

**Figure 8. Volcano plot:** **EA vs. PD-FMT**
**Table 3. Significantly Altered Acetylation Sites (EA vs. PD-FMT)**

| **Protein accession** | **Position** | **E/M Ratio** | **E/M P value** | **Regulated Type** | **Protein description** | **Gene name** |
| --- | --- | --- | --- | --- | --- | --- |
| O08992 | 120 | 3.236 | 0.0186198 | Up | Syntenin-1 | Sdcbp |
| O35381 | 110 | 2.372 | 0.0418884 | Up | Acidic leucine-rich nuclear phosphoprotein 32 family member A | Anp32a |
| P14211 | 143 | 2.711 | 0.0116772 | Up | Calreticulin | Calr |
| P16546 | 2421 | 2.568 | 0.0213383 | Up | Spectrin alpha chain, non-erythrocytic 1 | Sptan1 |
| P20152 | 292 | 2.981 | 0.0168192 | Up | Vimentin | Vim |
| P49025 | 1095 | 2.295 | 0.0260055 | Up | Citron Rho-interacting kinase | Cit |
| P54775 | 212 | 2.366 | 0.0137853 | Up | 26S proteasome regulatory subunit 6B | Psmc4 |
| Q04447 | 307 | 1.798 | 0.0169702 | Up | Creatine kinase B-type | Ckb |
| Q501J6 | 234 | 1.56 | 0.0385502 | Up | Probable ATP-dependent RNA helicase DDX17 | Ddx17 |
| Q64213 | 165 | 1.944 | 0.0124185 | Up | Splicing factor 1 | Sf1 |
| Q6NVF9 | 79 | 2.146 | 0.0477724 | Up | Cleavage and polyadenylation specificity factor subunit 6 | Cpsf6 |
| Q6PFR5 | 196 | 2.931 | 0.0154658 | Up | Transformer-2 protein homolog alpha | Tra2a |
| Q7TQF7 | 168 | 2.32 | 0.0126678 | Up | Amphiphysin | Amph |
| Q8BL66 | 746 | 1.931 | 0.0233429 | Up | Early endosome antigen 1 | Eea1 |
| Q8BMS1 | 759 | 1.574 | 0.0118339 | Up | Trifunctional enzyme subunit alpha, mitochondrial | Hadha |
| Q8K2B3 | 615 | 1.935 | 0.0311025 | Up | Succinate dehydrogenase [ubiquinone] flavoprotein subunit, mitochondrial | Sdha |
| Q9CQE1 | 57 | 1.701 | 0.0013714 | Up | Protein NipSnap homolog 3B | Nipsnap3b |
| Q9D819 | 128 | 2.149 | 0.0335276 | Up | Inorganic pyrophosphatase | Ppa1 |
| Q9DB20 | 162 | 1.798 | 0.0474203 | Up | ATP synthase subunit | Atp5po |
| Q9QYJ0 | 226 | 2.349 | 0.011949 | Up | DnaJ homolog subfamily A member 2 | Dnaja2 |
| Q9Z130 | 61 | 2.738 | 0.0119657 | Up | Heterogeneous nuclear ribonucleoprotein D-like | Hnrnpdl |
| Q9Z204 | 39 | 2.035 | 0.0456632 | Up | Heterogeneous nuclear ribonucleoproteins C1/C2 | Hnrnpc |
| Q9DBG3 | 131 | 0.596 | 0.0060308 | Down | AP-2 complex subunit beta | Ap2b1 |
| P05202 | 59 | 0.567 | 0.0035073 | Down | Aspartate aminotransferase, mitochondrial | Got2 |
| P05202 | 338 | 0.538 | 0.0431919 | Down | Aspartate aminotransferase, mitochondrial | Got2 |
| P14824 | 81 | 0.55 | 0.0364106 | Down | Annexin A6 | Anxa6 |
| P16546 | 2098 | 0.44 | 0.0203726 | Down | Spectrin alpha chain, non-erythrocytic 1 | Sptan1 |
| P17426 | 894 | 0.436 | 0.0428824 | Down | AP-2 complex subunit alpha-1 | Ap2a1 |
| P46096 | 369 | 0.655 | 0.0151535 | Down | Synaptotagmin-1 | Syt1 |
| P47754 | 268 | 0.439 | 0.0247393 | Down | F-actin-capping protein subunit alpha-2 | Capza2 |
| P53810 | 68 | 0.513 | 0.0307645 | Down | Phosphatidylinositol transfer protein alpha isoform | Pitpna |
| P60761 | 47 | 0.399 | 0.0402944 | Down | Neurogranin | Nrgn |
| P68040 | 185 | 0.613 | 0.019275 | Down | Receptor of activated protein C kinase 1 | Rack1 |
| P68254 | 49 | 0.649 | 0.0293272 | Down | 14-3-3 protein theta | Ywhaq |
| P70122 | 35 | 0.561 | 0.0039126 | Down | Ribosome maturation protein SBDS | Sbds |
| Q60631 | 20 | 0.473 | 0.0368423 | Down | Growth factor receptor-bound protein 2 | Grb2 |
| Q61301 | 119 | 0.63 | 0.0299853 | Down | Catenin alpha-2 | Ctnna2 |
| Q61699 | 656 | 0.384 | 0.0068166 | Down | Heat shock protein 105 kDa | Hsph1 |
| Q62261 | 1653 | 0.219 | 0.0245289 | Down | Spectrin beta chain, non-erythrocytic 1 | Sptbn1 |
| Q68FL4 | 191 | 0.638 | 0.0080104 | Down | Putative adenosylhomocysteinase 3 | Ahcyl2 |
| Q80VD1 | 245 | 0.571 | 0.0088414 | Down | Protein FAM98B | Fam98b |
| Q8BIG7 | 227 | 0.558 | 0.0403807 | Down | Catechol | Comtd1 |
| Q8BMF3 | 372 | 0.575 | 0.0449075 | Down | NADP-dependent malic enzyme, mitochondrial | Me3 |
| Q8C522 | 194 | 0.542 | 0.0365565 | Down | Endonuclease domain-containing 1 protein | Endod1 |
| Q91YM2 | 35 | 0.624 | 0.0104124 | Down | Rho GTPase-activating protein 35 | Arhgap35 |
| Q99PJ0 | 81 | 0.587 | 0.0422875 | Down | Neurotrimin | Ntm |
| Q9D1A2 | 383 | 0.555 | 0.0360558 | Down | Cytosolic non-specific dipeptidase | Cndp2 |
| Q9EPU0 | 373 | 0.385 | 0.0138964 | Down | Regulator of nonsense transcripts 1 | Upf1 |
| Q9ESM3 | 168 | 0.627 | 0.0303995 | Down | Hyaluronan and proteoglycan link protein 2 | Hapln2 |

**Gene Ontology (GO) Enrichment Analysis**

GO analysis covered three domains:

**PD-FMT vs. NC DAP-associated proteins (DAAPs)**:

Biological Process: Cellular process, localization, biological regulation, response to stimulus, metabolic process, signaling, multicellular organismal process, developmental process, multi-organism process, immune system process (Fig. 9)

Cellular Component: Intracellular, cell, protein-containing complex

Molecular Function: Binding, catalytic activity, structural molecule activity, transporter activity, molecular function regulator

**EA vs. PD-FMT DAAPs**:

Biological Process: Cellular process, biological regulation, metabolic process, multicellular organismal process, response to stimulus, localization, developmental process, signaling, multi-organism process, immune system process, reproduction, locomotion (Fig. 10)

Cellular Component: Intracellular, cell, protein-containing complex

Molecular Function: Binding, catalytic activity, structural molecule activity, transporter activity, transcription regulator activity, molecular transducer activity, protein folding chaperone


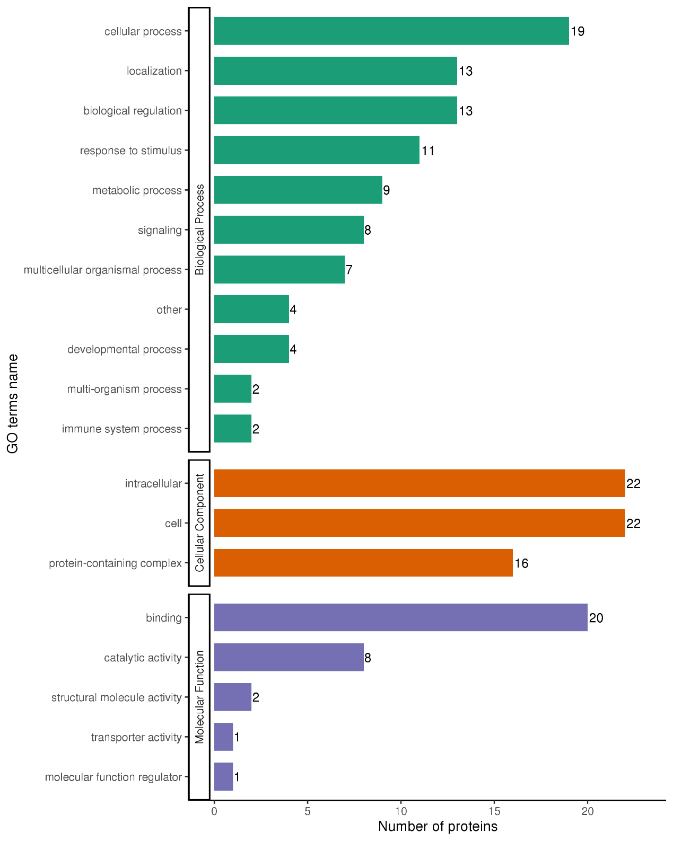


**Figure 9. GO Analysis of DAAPs (PD-FMT vs. NC)**


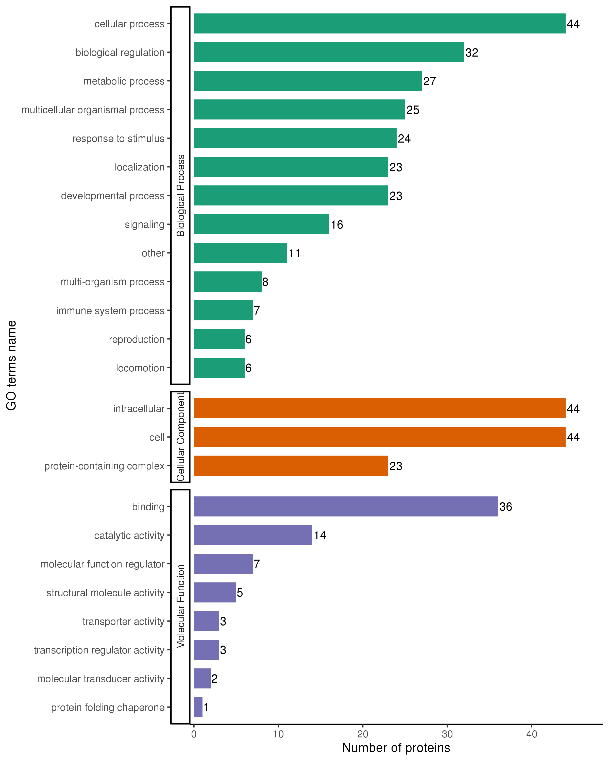

**Figure 10. GO Analysis of DAAPs (EA vs. PD-FMT)**

**Clusters of Orthologous Groups (COG) Classification**

PD-FMT vs. NC DAAPs: Secondary metabolites biosynthesis/transport/catabolism, nucleotide transport/metabolism, energy production/conversion, replication/recombination/repair, translation/ribosomal structure/biogenesis, RNA processing/modification, intracellular trafficking/secretion/vesicular transport, signal transduction mechanisms, posttranslational modification/protein turnover/chaperones, cytoskeleton, cell cycle control/cell division/chromosome partitioning (Fig. 11).

EA vs. PD-FMT DAAPs: Energy production/conversion, lipid transport/metabolism, amino acid transport/metabolism, secondary metabolites biosynthesis/transport/catabolism, coenzyme transport/metabolism, RNA processing/modification, transcription, replication/recombination/repair, translation/ribosomal structure/biogenesis, signal transduction mechanisms, cytoskeleton, intracellular trafficking/secretion/vesicular transport, posttranslational modification/protein turnover/chaperones, extracellular structures (Fig. 12).


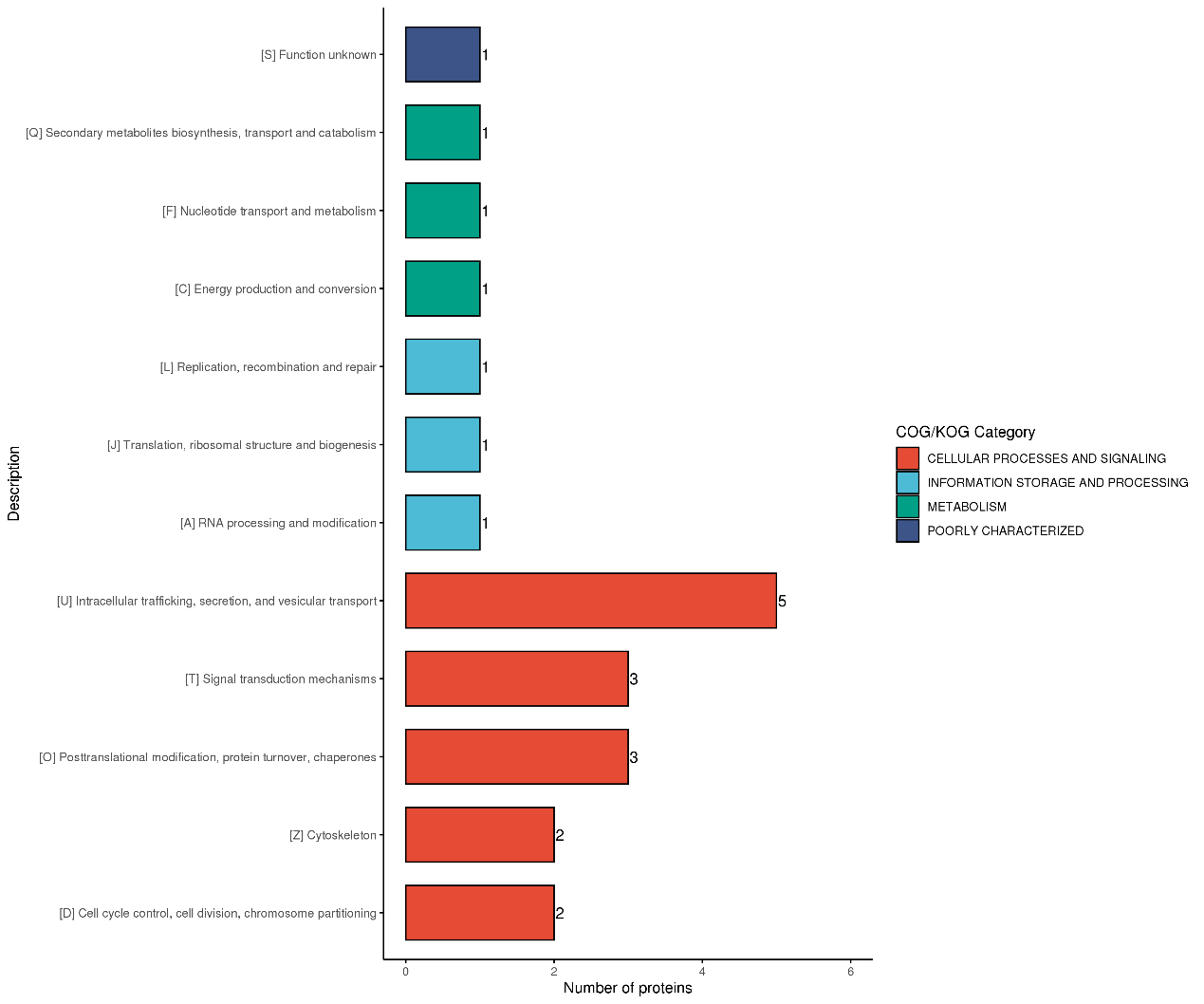


**Figure 11. COG Analysis of DAAPs (PD-FMT vs. NC)**


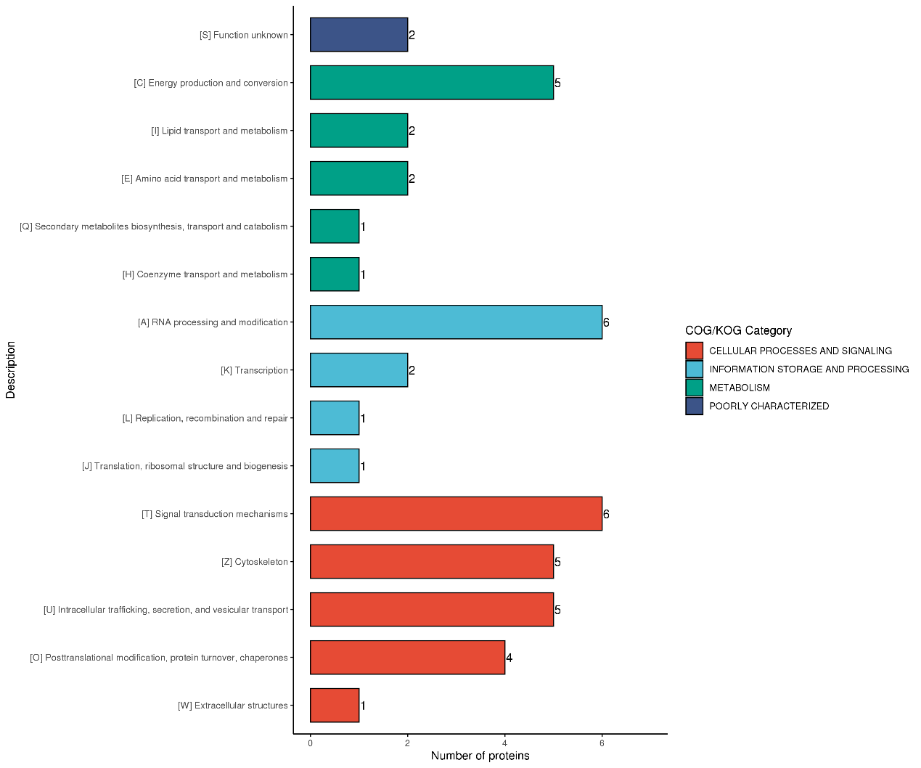

**Figure 12. COG Analysis of DAAPs (EA vs. PD-FMT)**

**KEGG Pathway Enrichment**

PD-FMT vs. NC: Pyrimidine metabolism, toxoplasmosis, cell cycle, Hippo signaling pathway (Fig. 13).

EA vs. PD-FMT: Arginine and proline metabolism, beta-Alanine metabolism, synaptic vesicle cycle, endocytosis (Fig. 14).


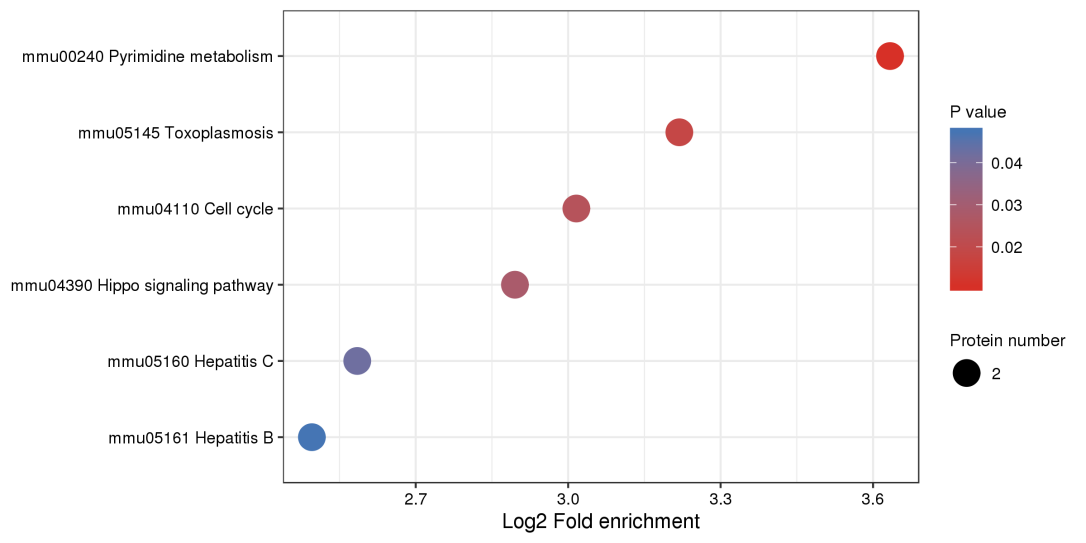


**Figure 13. KEGG Pathway Enrichment (PD-FMT vs. NC)**


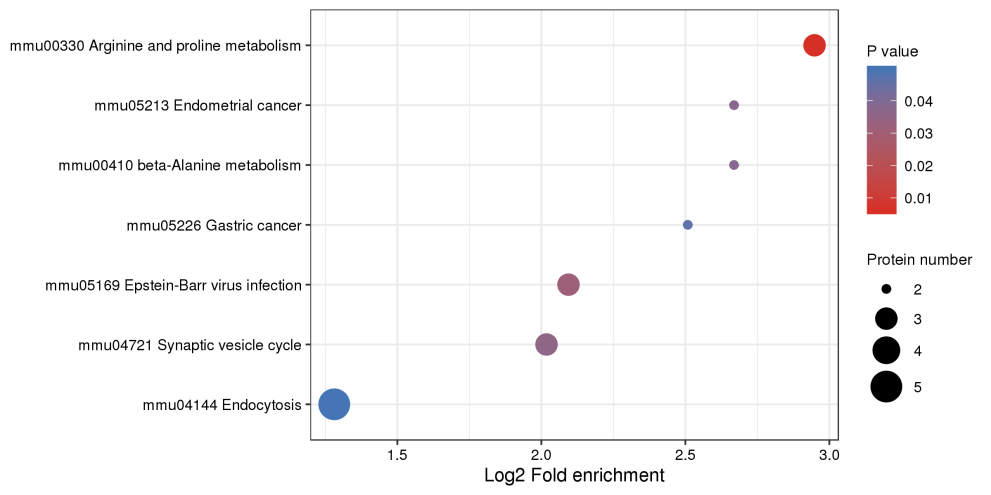

**Figure 14. KEGG Pathway Enrichment (EA vs. PD-FMT)**

**Intersection Analysis and Validation**

To identify potential targets of electroacupuncture (EA) intervention in a PD mouse model induced by fecal microbiota transplantation from Parkinson’s disease patients (PD-FMT), we performed 4D label-free acetylproteomic analysis on brain tissue proteins from normal control (NC), PD-FMT, and EA-treated mice. Data processing included acetylated peptide identification, reproducibility evaluation, quality control, screening of differentially acetylated proteins, along with Gene Ontology (GO), Clusters of Orthologous Groups (COG), and KEGG pathway enrichment analyses. Differentially acetylated sites between groups were identified based on a fold change (FC) threshold of >1.5 for upregulation and <1/1.5 for downregulation, with a significance level of *P* < 0.05. Volcano plots revealed 16 significantly upregulated and 7 downregulated acetylation sites (corresponding to 16 and 7 proteins, respectively) in the PD-FMT group compared to the NC group (Fig. 2A). In the EA group versus the PD-FMT group, 22 sites were upregulated and 27 downregulated (affecting 22 and 26 proteins, respectively) (Fig. 2B). Intersection analysis of differentially acetylated proteins from the NC vs. PD-FMT and PD-FMT vs. EA comparisons identified three overlapping targets: Sptan1, Ywhaq, and Upf1 (Fig. 2C, D). Acetylation levels of Sptan1 and Ywhaq were significantly increased in the substantia nigra of PD-FMT mice compared to NC controls (both *P* < 0.01), and EA treatment significantly reduced their acetylation relative to the PD-FMT group (*P* < 0.01 and *P* < 0.05) (Fig. 2E–G). Based on literature evidence, Ywhaq was selected for further validation by Western blot. Consistent with the proteomic results, Ywhaq acetylation was significantly elevated in both the HC-FMT and PD-FMT groups compared to the NC group (*P* < 0.01), and EA treatment significantly decreased its acetylation compared to the PD-FMT group (*P* < 0.05) (Fig. 2H, I).


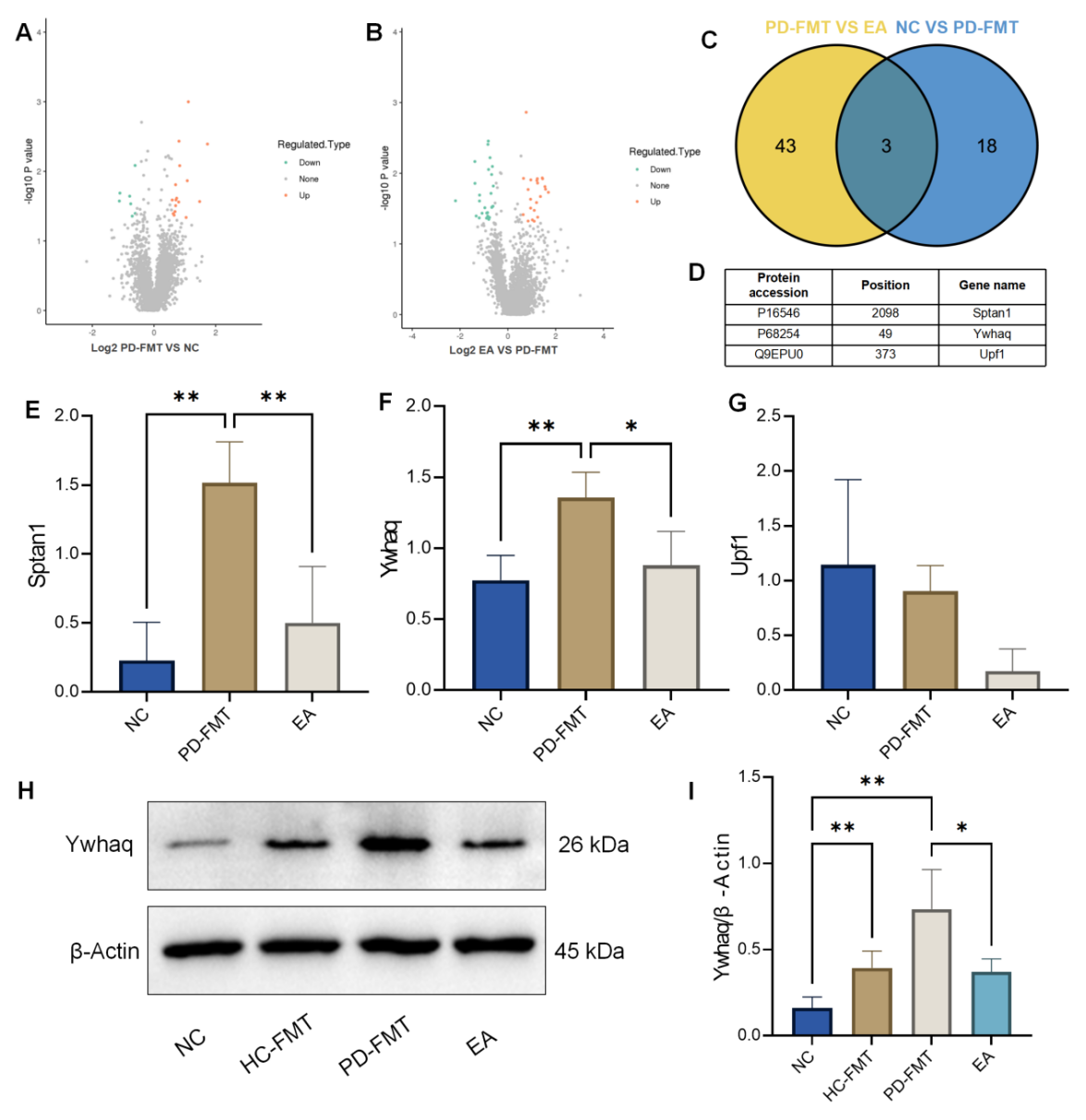


**Figure 2. Effects of Electroacupuncture on Protein Acetylation in the Substantia Nigra of PD Mice**

*Notes:*

1. Volcano plot: PD-FMT vs. NC
   (B) Volcano plot: EA vs. PD-FMT
   (C) Venn diagram of overlapping DAPs
   (D) Heatmap of intersecting acetylated proteins
   (E-G) Acetylation levels of Sptan1/Ywhaq/Upf1 (mean ± SEM)
   (H) Representative WB bands for acetyl-Ywhaq
   (I) Quantification of acetyl-Ywhaq/β-actin ratio
   **P*<0.05, ***P*<0.01, n=4.
