# Supplementary figures and images for: Electroacupuncture alleviates Parkinson’s disease by targeting HDAC/SIRT-mediated deacetylation of 14-3-3
Source: Front Aging Neurosci. 2026 Jan 14;17:1719326. doi: 10.3389/fnagi.2025.1719326 (PMC12847356; doi:10.3389/fnagi.2025.1719326)

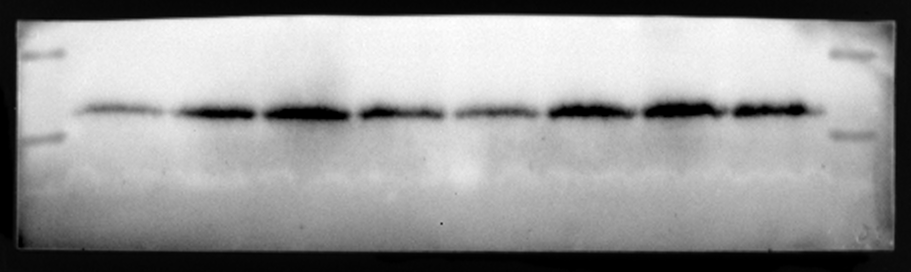

Supplement: Supplementary file 3 [file Data_Sheet_1.zip › western blot images in .tif/Alpha-synuclein/Alpha-synuclein.Tif]

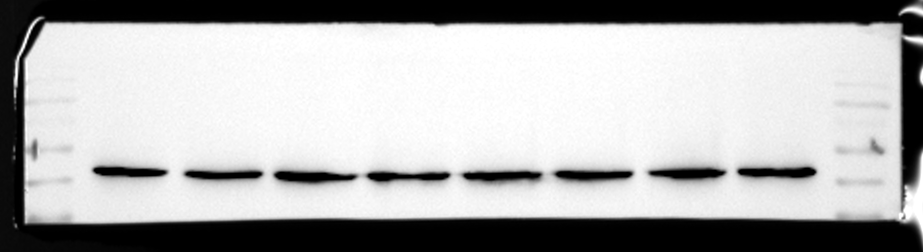

Supplement: Supplementary file 3 [file Data_Sheet_1.zip › western blot images in .tif/Alpha-synuclein/β actin(Alpha-synuclein).Tif]

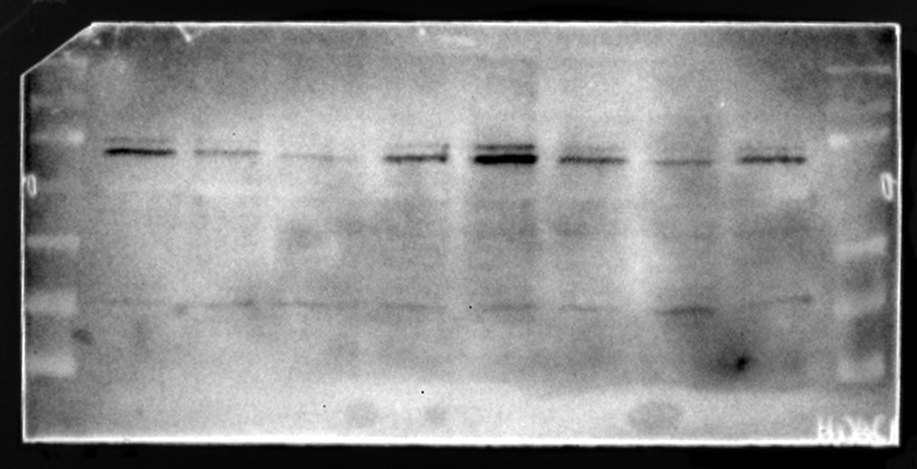

Supplement: Supplementary file 3 [file Data_Sheet_1.zip › western blot images in .tif/HDAC1/HDAC1.Tif]

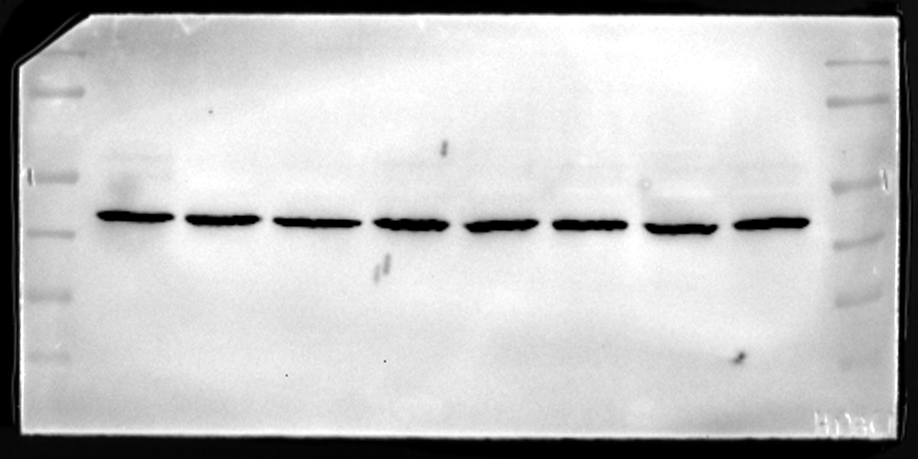

Supplement: Supplementary file 3 [file Data_Sheet_1.zip › western blot images in .tif/HDAC1/β Actin(HDAC1).Tif]

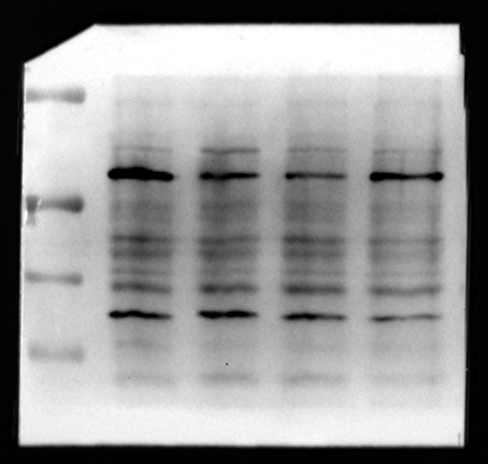

Supplement: Supplementary file 3 [file Data_Sheet_1.zip › western blot images in .tif/HDAC2/HDAC2.Tif]

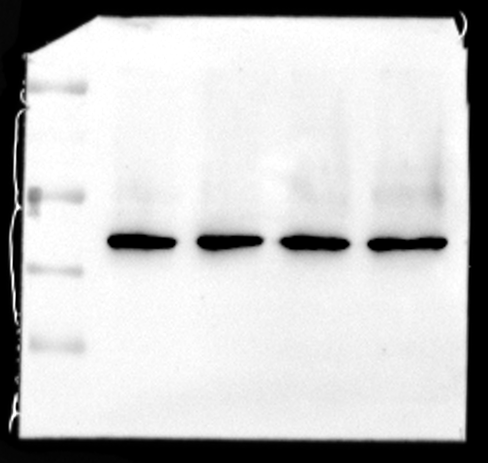

Supplement: Supplementary file 3 [file Data_Sheet_1.zip › western blot images in .tif/HDAC2/β Actin(HDAC2).Tif]

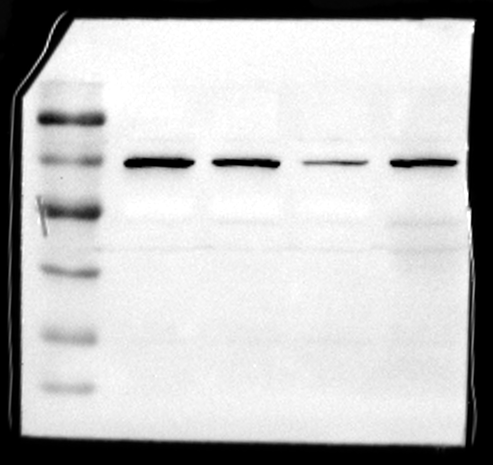

Supplement: Supplementary file 3 [file Data_Sheet_1.zip › western blot images in .tif/HDAC3/HDAC3.Tif]

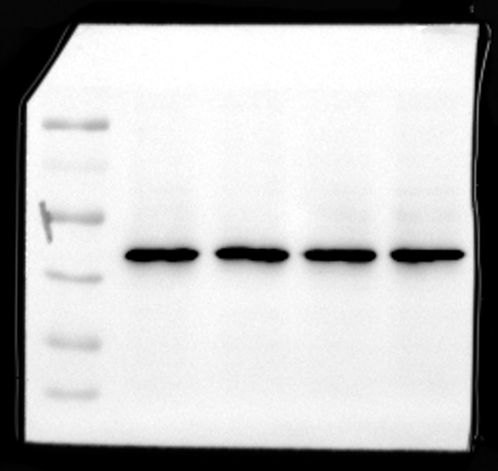

Supplement: Supplementary file 3 [file Data_Sheet_1.zip › western blot images in .tif/HDAC3/β Actin(HDAC3).Tif]

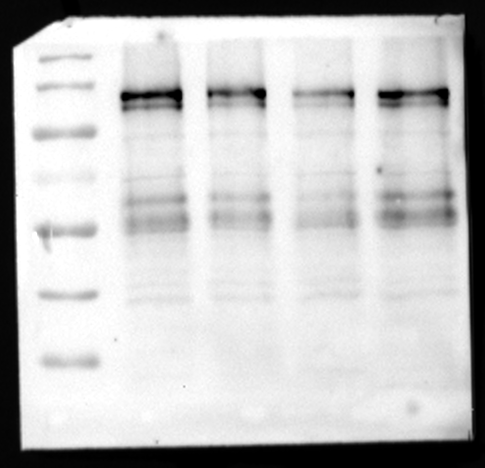

Supplement: Supplementary file 3 [file Data_Sheet_1.zip › western blot images in .tif/SIRT1/SIRT1.Tif]

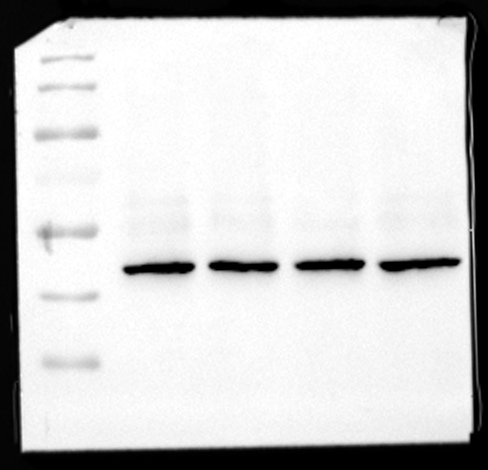

Supplement: Supplementary file 3 [file Data_Sheet_1.zip › western blot images in .tif/SIRT1/β Actin(SIRT1).Tif]

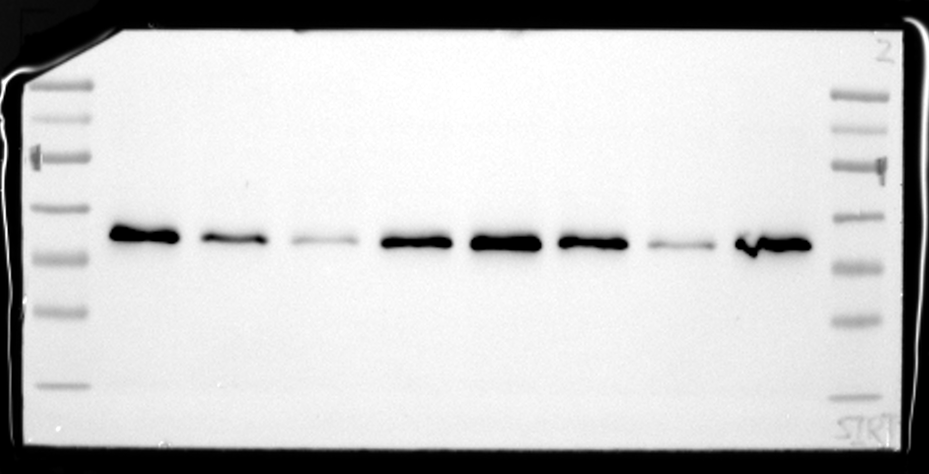

Supplement: Supplementary file 3 [file Data_Sheet_1.zip › western blot images in .tif/SIRT2/SIRT2.Tif]

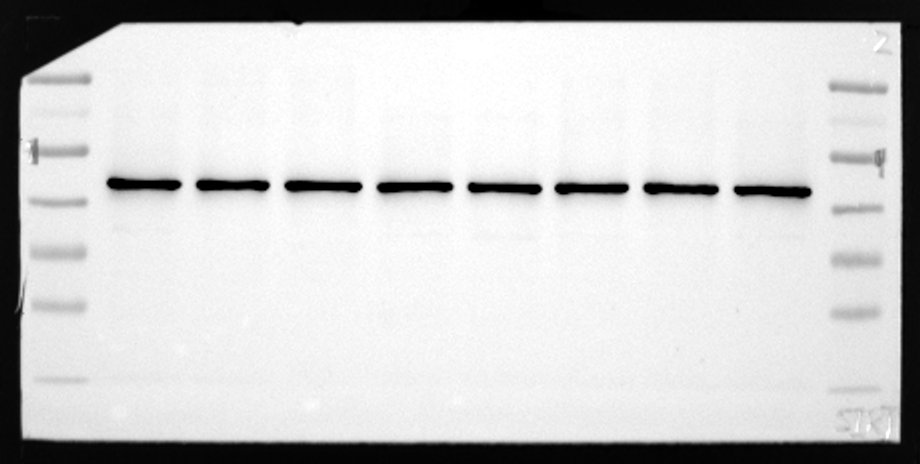

Supplement: Supplementary file 3 [file Data_Sheet_1.zip › western blot images in .tif/SIRT2/β Actin(SIRT2)..Tif]

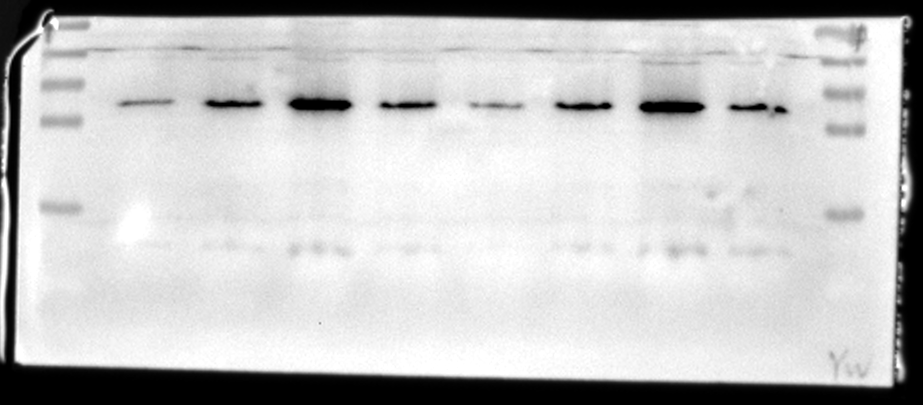

Supplement: Supplementary file 3 [file Data_Sheet_1.zip › western blot images in .tif/Ywhaq/Ywhaq.Tif]

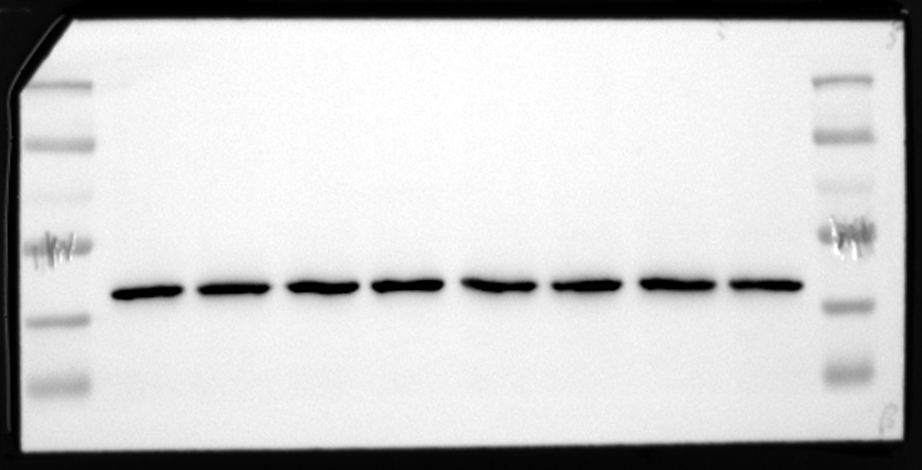

Supplement: Supplementary file 3 [file Data_Sheet_1.zip › western blot images in .tif/Ywhaq/β Actin.Tif]
